# The High Expression of p53 Is Predictive of Poor Survival Rather *TP53* Mutation in Esophageal Squamous Cell Carcinoma

**DOI:** 10.1155/2023/3801526

**Published:** 2023-01-10

**Authors:** Yan Jin, Xueke Zhao, Xin Song, Ran Wang, Zongmin Fan, Panpan Wang, Miaomiao Yang, Fuyou Zhou, Qide Bao, Lidong Wang

**Affiliations:** ^1^State Key Laboratory of Esophageal Cancer Prevention & Treatment and Henan Key Laboratory for Esophageal Cancer Research of The First Affiliated Hospital, Zhengzhou University, Zhengzhou, China; ^2^Department of Human Anatomy & Histoembryology, School of Basic Medical Sciences, Xinxiang Medical University, Xinxiang, China; ^3^Department of Thoracic Surgery, Anyang Tumor Hospital, Anyang, China; ^4^Department of Oncology, Anyang District Hospital of Puyang City, Anyang, China

## Abstract

*TP53* is a well-known tumor suppressor gene and one of the most common genetic alterations in human cancers. However, the role of p53 as a prognostic marker of esophageal squamous cell carcinoma (ESCC) is controversial in the association between *TP53* alterations and clinical outcomes. To address this issue, we evaluated *TP53* mutations, p53 protein expression, clinicopathological parameters, and survivals rates in a large scale of patients with ESCC. Two cohorts were included in this study: *TP53* mutations were detected by next-generation sequencing in 316 ESCC patients, and p53 protein expression was tested by immunohistochemistry in 6,028 ESCC patients. Survival analysis was performed using the Kaplan–Meier curve and the Cox proportional hazards model. *TP53* mutations were found in ESCC patients from 241 of 316 (76.3%), and the rate of positive expression of p53 protein was 59.1% in 6,028 ESCC patients (including 1819 with high expression of p53 protein), respectively. Most mutations were missense, which has a high expression of p53 protein. Compared with wild-type*TP53*, *TP53* gene mutations were not significantly associated with survival time (*p*=0.083). In multivariate analysis, the p53 protein expression was an independent prognostic factor for ESCC. The high-expression group of p53 protein has poor survival (*p* < 0.001) compared to low-expression group in patients with ESCC. The high expression of the p53 protein, not the *TP53* mutation, is predictive of poor survival in patients with ESCC, and p53 protein expression might have the potential to be a prognosis biomarker and therapy target in ESCC.

## 1. Introduction

Esophageal carcinoma is one of the most aggressive cancers and the sixth leading cause of cancer death [1]. Esophageal cancer (EC) is the fourth most common malignancy associated with cancer-related death, and esophageal squamous cell carcinoma (ESCC) is the most common pathological subtypes (>90%) in China [2–5]. With limited early clinical diagnosis approaches and few targeted therapies, although the five-year survival rate has improved during past decades, it still remains dismal, only at 10%–30% in most countries [6]. The most effective way to improve the survival rate of ESCC patients is through early detection and treatment. Therefore, the discovery of molecular markers for early screening, prognosis, and efficacy of evaluation to provide personalized treatment for ESCC patients is one of the entry points to reduce the incidence and improve the survival rate of patients with ESCC.

*TP53* is one of the most frequently mutated genes in human cancers and has occurred in more than 50% of all human cancers [7, 8]. A recent study analyzed the *TP53* mutational spectra of 7,525 pan-cancer tissues and found *TP53* mutations in 35% of all 30 tumor-type samples, of which the most mutated cohorts are more than 80% and the lowest percentage of *TP53* mutation was less than 1% [9]. Even in the same tumor, the heterogeneity including differences in histology, molecular subtype, pathogenic factors, tumor stage, and degrees of differentiation can also affect the evaluation of the frequency of *TP53* mutations [10]. Majority mutations of *TP53* cause the loss of function (LOF) of wild-type*TP53* and abrogate their ability to bind on specific DNA motif and perform its tumor-suppressive function. Gain of function (GOF), dominant negative effect on the wild-type*TP53* allele, the loss of heterozygosity of *TP53*, and interactions with viral proteins also result in the activity loss of wild-type*TP53* [11, 12].

Recent studies using whole-genome and exome sequencing in patients with EC revealed that the most mutated gene is *TP53*, which means that at least *TP53* has a significant influence on EC pathogenicity [13, 14]. In general, *TP53* mutations often cause changes on the amino acid sequence of the p53 protein, thus disrupting the function of p53 for tumor inhibition. Under normal conditions, it is difficult to detect the expression of wild-type p53 protein due to its short half-life. Therefore, we usually detect the expression level of p53 protein (mutant-type) using immunohistochemistry (IHC) in clinical diagnosis, but in ESCC, the positive rate of p53 protein expression varies greatly in different reports, ranging from 27 to 75% [15]. At present, the role of p53 as a prognostic marker of ESCC is controversial. To date, the mechanism and correlation between the expression of the p53 protein and mutation of the *TP53* gene or prognostic effect in ESCC are limited and contradictory. The clinical value of *TP53* mutation and p53 protein expression is worth further exploration.

Herein, we conducted an analysis of *TP53* mutation and p53 protein expression in two-large ESCC cohorts, using a series of methods including whole-genome sequencing (WGS), whole exon sequencing (WES), regionally targeted sequencing (TRS) and IHC, to further clarify the association between *TP53* mutation status/ the expression of p53 protein and clinicopathologic phenotype and prognosis. This study provided effective and reliable evidence showing *TP53* alterations as a biomarker for clinical diagnosis and treatment in ESCC.

## 2. Materials and Methods

### 2.1. Patients and Samples

**Figure 1:**
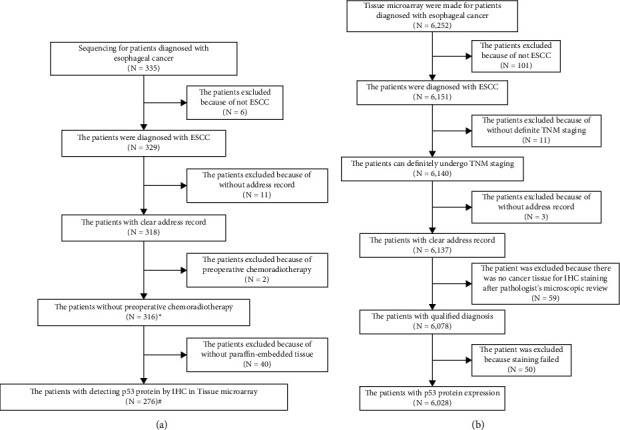
Flow chart of patient selection. Patients who had *TP53* mutations by sequencing (a) and p53 protein expression by IHC (b) were included on the basis of clinical and histopathological characteristics and survival status. ^*∗*^ the *TP53* mutation analysis was included in 316 patients. ^#^ the p53 protein expression analysis was included in 276 patients.

All of the patients and samples were selected from the tissue bank and database of about 500,000 esophageal and gastric cardia carcinomas (1973–2020), established by the State Key Laboratory of Esophageal Cancer Prevention & Treatment and the Henan Key Laboratory for Esophageal Cancer Research of The First Affiliated Hospital, Zhengzhou University [16–18]. The database was reviewed to select the present study cohort. All medical records were collected, including detailed clinical, pathological, and survival information. Carcinoma and adjacent noncancerous tissues of 335 patients, which included frozen tissue samples and paraffin tissue samples (for using WGS, WES, and TRS) were collected after surgery. Postoperative tumor paraffin-embedded tissues from 6,252 patients were used for producing tissue microarray (TMA). During the cases of screening, patients were excluded from the database according to the following standard: non-ESCC patients, lacking T, N, M stage, preoperative treatment, incomplete following-up, without tissue for IHC staining in TMA, or failed staining. The detailed flow chart for patient enrollment is shown in Figure 1.

For the first cohort, a total of 316 surgically resected ESCC tissue specimens (paired primary malignant and adjacent normal) were collected. The tissues were analyzed on a different platform: 316 paired primary malignant and adjacent normal tissues, including 19 cases with frozen tissues that had WGS data available; 92 samples (51 cases of frozen tissue and 41 cases of paraffin-embedded tissues) that had WES data available; and 205 samples (203 cases of frozen tissue and 2 cases of paraffin-embedded tissues) had TRS data available. Among the 316 patients, 276 were used to make TMA. The remaining 40 had no tumor specimen available (owing to a lack of specimen or insufficient tumor cells). The second cohort that contains 6,028 from the 6,252 patients with ESCC TMA was included in the final statistical analysis.

All the patients enrolled for this study were staged using the Union for International Cancer Control (UICC) staging standards, 6th (2002), for esophageal cancer. All patients were followed up after diagnosis until the date of death or December 2019.

### 2.2. Genomic DNA Extraction

Before DNA extraction, tissue was stained with hematoxylin and eosin (H&E) in order to assess the accurate histopathology for each case. The samples confirmed by a pathologist and in which tumor cells accounted for ≥50%, were chosen for DNA extraction. DNA was isolated from frozen tissue and formalin-fixed, paraffin-embedded (FFPE) tissue. Frozen tissue specimens were collected during surgery, snap-frozen in liquid nitrogen, and stored at −80°C. DNA was extracted from frozen tissue using the phenol-chloroform protocol [19]. For FFPE tissue, DNA was extracted from 8 sections with a 10 *μ*m thickness of each paraffin block using the QIAamp DNA FFPE tissue kit (Qiagen, Hilden, Germany).

### 2.3. Detection of *TP53* Mutation by Next Generation Sequencing

Extracted genomic DNA was examined on agarose gel and by Nanodrop 2000 and Qubit 2.0. Mutations of *TP53* were determined using NGS, including WGS, WES, and TRS. DNA library was constructed and captured using TruSeq Nano DNA HT Sample Prep Kit for WGS sequencing, and Agilent SureSelect Human All Exon V6 for WES sequencing, and the SureSelect XT2 Target Enrichment System for the Illumina Multiplexed Sequencing Platform for TRS sequencing following the manufacturer's recommendations, and then sequenced by an Illumina HiSeqPE150 or Illumina HiSeq 2500 sequencing platform (collaborating with Novogene Bioinformatics Technology Co., Tianjin, China).

### 2.4. Immunohistochemical Staining for p53 Protein Expression

**Figure 2:**
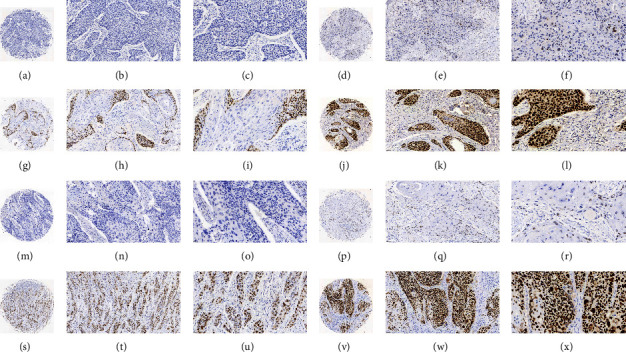
Immunohistochemical staining scoring criteria for p53 protein in ESCC (A, D, G, J, M, P, S, V, magnification ×80, B, E, H, K, N, Q, T, W, magnification ×200, C, F, I, L, O, R, U, X, magnification ×400). (a–l) Scoring criteria of pattern of immunostaining: (a–c) entirely negative staining; (d–f) scattered; (g–i) focal; (j–l) diffuse. (m–x) Scoring criteria of intensity of immunostaining: (m–o) negative staining; (p–q) weak staining; (s–u) moderate staining; (v–x) strong staining.

IHC staining of 4 *μ*m TMA sections was performed by a 2-step protocol using a p53 antibody (1 : 100 dilution) and DAB detection kit (both from Wuhan Servicebio Technology Co., Wuhan, China). In each experiment, both positive and negative controls were included. All images were captured by CaseViewer 2.2 for Windows (3DHISTECH, Budapest, Hungary, Figure 2).

### 2.5. Establishment of Scoring Criterion for p53 Immunohistochemical Staining

The scoring of IHC staining was completed independently by two experienced pathologists. Staining location, intensity, and patterns were reviewed. Tumor cells having a dark brown precipitate in their nuclei were the criterion for a positive reaction. The intensity of staining was grouped into four grades: 0 = entirely negative; 1 = weak; 2 = moderate; 3 = strong. The immunostaining patterns were divided into four terms: 0 = entirely negative; 1 = scattered, meaning only some isolates were positive cells; 2 = focal, where clusters of positive cells were seen in some areas; 3 = diffuse, in which the sheets of positive cells were found throughout most of the areas (Figure 2) [20]. The final results were multiplied by the scores of the immunostaining patterns and staining intensity. Patients were categorized as “high expression (>4)” or “low expression (0∼4)” by the use of IHC scoring criteria.

### 2.6. Statistical Analyses

Statistical analysis was processed by SPSS for Windows, version 25.0. The *T*-test and chi-square test or Fisher exact test were used to compare the association of categorical and continuous variables between different groups, respectively. The Kaplan–Meier method analyzed survival tendency and used the log-rank test to compare the survival curves. Cox proportional-hazard models were used for the univariate and multivariate analyses to estimate the hazard ratio of each clinicopathological feature for overall survival (OS). All predictors with *p* value <0.1 in univariate Cox were selected in multivariate Cox analysis. *p* values were 2-tailed and considered statistically significant with less than 0.05.

## 3. Results

### 3.1. The Clinicopathological Distributions of ESCC Patients Results

**Figure 3:**
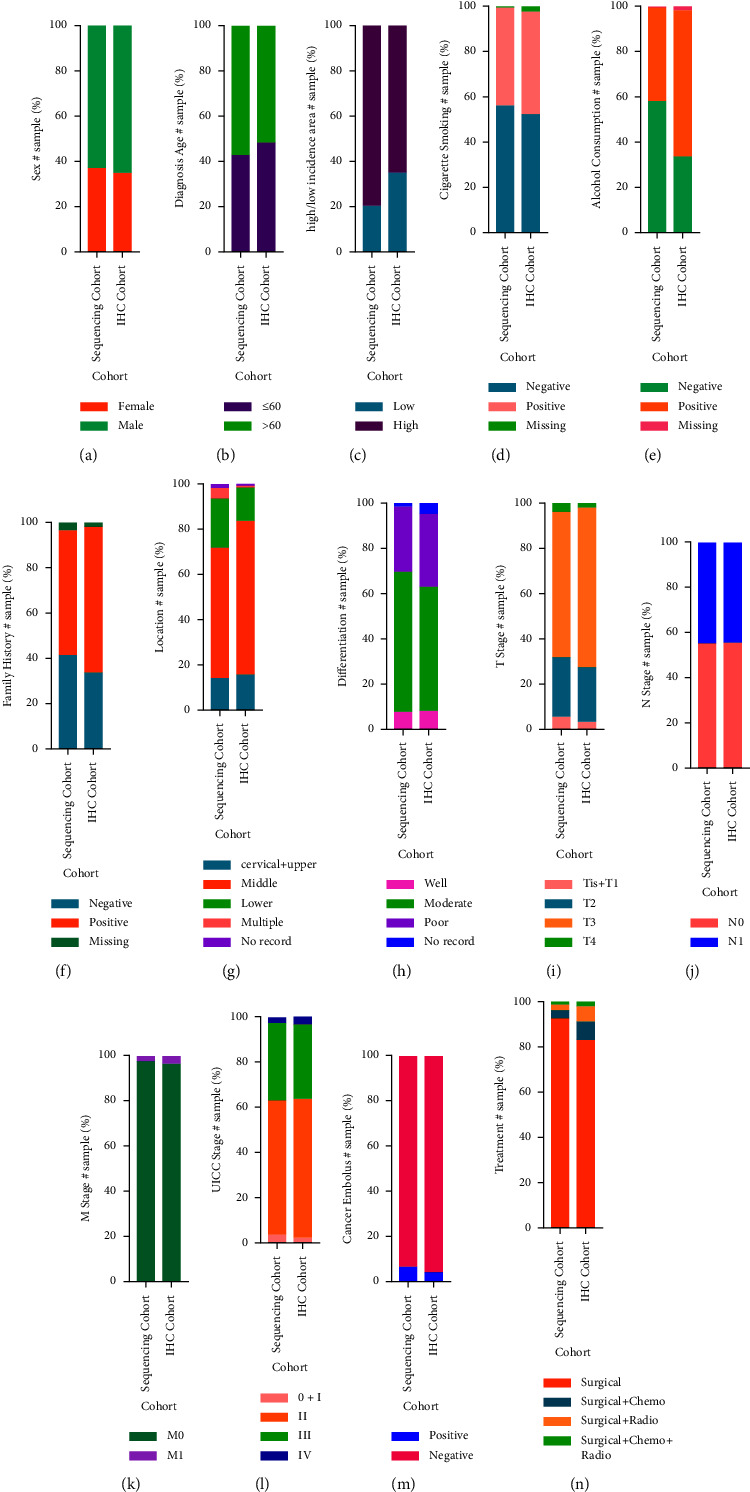
Baseline characteristics of patients with ESCC were included in both study cohorts. The proportions of the two study cohorts are shown according to sex (a), age at diagnosis (b), high/low incidence area (c), cigarette smoking (d), alcohol consumption (e), family history (f), location (g), differentiation (h), T stage (i), N stage (j), M stage (k), UICC stage (l), cancer embolus (m), and treatment (n), respectively.

To discover the genetic alteration of *TP53* in ESCC, we collected tumor samples to set up two cohorts, including 316 patients for sequencing analysis (276 patients out of 316 patients performed IHC to detect the expression of p53 protein), and 6,028 patients for the expression of p53 protein in TMA, for investigation in this study (Figure 1). Detailed clinicopathological data are listed in Figure 3 and Table 1. The follow-up period of all those patients ranged from 0.08 years to 30.87 years, and the median survival time was 2.98 years.

### 3.2. Distribution of *TP53* Somatic Mutations and p53 Protein

**Figure 4:**
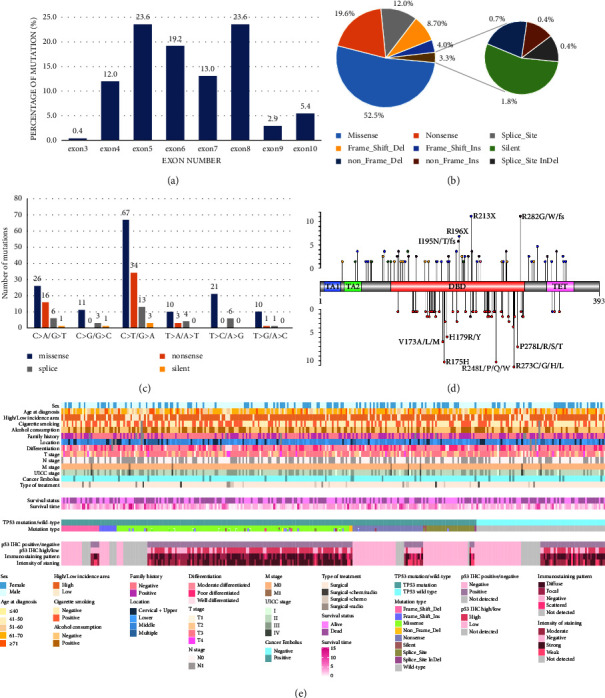
Type of somatic *TP53* mutation in ESCC patients. (a) Distribution of *TP53* mutations according to the affected exons. (b) Pie chart showing the proportion of the different types of somatic *TP53* mutations. (c) Different types of mutations in the spectrum of *TP53* mutations. (d) Graphical representation of the site of mutations in the coding sequence of *TP53*: red lollypop symbols in the lower panel representing missense mutations, blue, yellow, green, pink, and purple lollypop symbols in the upper panel representing others mutations (including nonsense, frameshift del, frameshift ins, silent, mixed type, respectively). Mutations occurring six or more are labeled: TA1, TA2, transactivation domain; DBD, DNA binding domain; TET, tetramerization domain. The figure was generated by the Illustrator for biological sequences version 1.0. (e) Summary of patients' characteristics and alterations for *TP53* and p53 protein in ESCC. Each column represents one patient sample. The phenotypic information in 316 ESCC patients is shown in the upper panel. Alterations for the *TP53* gene and p53 protein are shown in the bottom panel.

We evaluated *TP53* somatic alterations in the first cohort, and the results showed that 241 of the 316 ESCC patients (76.3%) exhibited a total of 276 *TP53* somatic mutations, which included 208 cases having only one mutation and 33 having multiple (two and three) mutations (Tables 2 and 3). *TP53* mutations were mainly located in the exon 5–8 (79.4%), meaning that most mutations occurred in the DBD, and very few mutations occurred in the AD1, AD2, and TET domains. Mutations were mostly clustered in exon 5 (23.6%) and exon 8 (23.6%), followed by exon 6 (19.2%) (Figure 4(a)**)**.

Next, we investigated the overall pattern of the 276 somatic mutations identified in the *TP53*, in which 145 were missense (52.5%), 54 were nonsense (19.6%), 33 were splices (12.0%), 24 were frameshift deletion (8.7%), eleven were frame-shit insertion (4.0%), five were silent (1.8%), two were nonframe deletion (0.7%), one was nonframe insertion (0.4%), and one was splice site insertion and deletion (0.4%) (Figure 4(b) and Table 4). For the protein domain distribution of missense mutations, the majority of which occurred in the DBD domain. However, the main type of mutation varied with different exons. In exons 5, 7, and 8, missense mutations accounted for more than 70% (73.8%, 75.0%, and 70.7%, respectively).

We observed that the proportions of the *TP53* mutation spectrum were 49/237 (20.7%) for *C* > *A*/*G* > *T*, 15/237 (6.3%) for *C* > *G*/*G* > *C*, 117/237 (49.4%) for *C* > *T*/*G* > *A*, 17/237 (7.2%) for *T* > *A*/*A* > *T*, 27/237 (11.4%) for *T* > *C*/*A* > *G*, 12/237 (5.1%) for *T* > *G*/*A* > *C*. The transition was predominant (144/237, 60.8%), followed by transversion (93/237, 39.2%) (Figure 4(c) and Table 4).

Notably, we found the frequency of nine protein mutations of p53 protein, including four hotspot mutations (p.R175, p.R248, p.R273, and p.R282) and five nonhotspot mutations (p.V173, p.H179, p.R196, p.R213, and p.P278) [21, 22], were more than five, respectively. Hotspot mutations comprised 29.0% (42/145) of all p53 missense mutations (Figure 4(d)).

### 3.3. Correlation of p53 Protein Expression with TA Class and Align GVGD Classifications

To speculate on the effect of the protein function of the *TP53* missense mutation sites, we submitted queries to the IARC *TP53* Database (https://p53.iarc.fr) [23–25]. According to TA classification, 145 *TP53* missense mutation sites were divided into three categories: functional (4/145, 2.7%), partially functional (11/145, 7.6%), and not-functional (130/145, 89.7%) based on the TA class of the protein function (Table 4). We further observed that four of the 145 sites (in 135 patients) with missense mutations didn't affect the function of the wild-p53 protein. However, only one of the four sites was a single mutation, and its p53 protein expression was negative. The other three sites were multiple mutations, and their p53 protein expression was positively detected (two were high, and one was low). Among the 130 sites (126 patients) with not-functional (108 patients with p53 protein expression tests), 105 patients were positive p53 protein expression (105/108, 97.2%), including 74 patients with p53 protein high expression (74/108, 68.5%). The p53 protein expression level is consistent with the predicted function based on the mutation sites. In the Align GVGD classification, we did not observe a significant difference in the positive rate of p53 protein in each group.

### 3.4. Association between *TP53* Mutation and Protein Expression with Clinicopathological Parameters and Survival in ESCC

**Figure 5:**
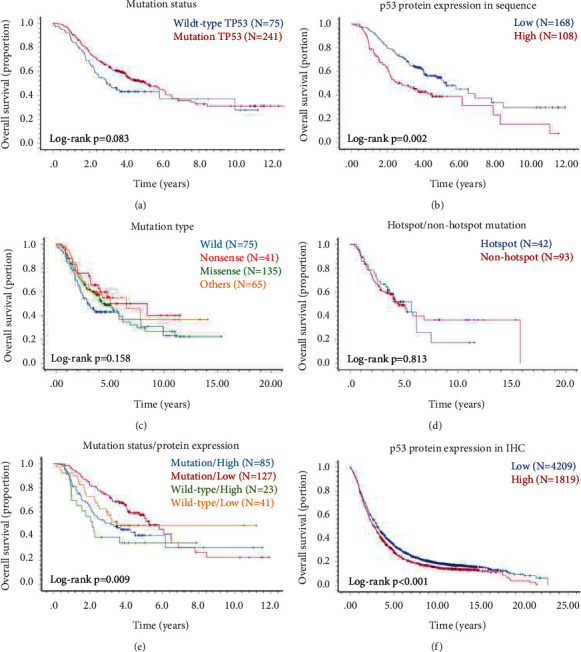
Kaplan–Meier curves survival analysis of ESCC patients with different *TP53* mutations and p53 protein expression. (a) OS of *TP53* mutation and wild-type in 316 patients with sequencing data. (b) OS of p53 protein high and low expression in 276 patients who have *TP53* mutation and p53 protein expression. (c) OS of different types of mutation in 316 patients with sequencing data. (d) OS of nonhotspot and hotspot mutation in 135 patients who have missense mutation. (e) OS of different groups of *TP53* mutation status/p53 protein expression in 276 patients who have *TP53* mutation and p53 protein expression. (f) OS of p53 protein high and low expression in 6,028 patients who have p53 expression in TMAs.

In the first cohort, we did not find any remarkable association between the *TP53* mutation and these clinicopathological characteristics in ESCC (Table 2). Although there was no statistically significant difference in survival time when comparing the patients with and without *TP53* mutations (Figure 5(a)), surprisingly, the results showed that the patients who had high protein expression of p53 exhibited significantly worse survival than those with low expression when the population narrow down to 276 patients (*p*=0.002, Figure 5(b)). The median survival time for 108 patients with high protein expression of p53 and for 168 patients who had low expression was 2.79 ± 0.63 and 5.27 ± 0.52 years, respectively. In addition, no significant difference was observed in the survival time of different mutation types of *TP53* (*p* > 0.05, Figure 5(c)), and no significant difference was observed in survival time between the hotspot and nonhotspot in *TP53* missense mutations patients (*p* > 0.05, Figure 5(d)).

To better understand the factors which contribute to the association between *TP53* mutations/high p53 expression in patients and survival, we compared the mutation types of p53 patients with ESCC. The high expression of the p53 protein was 66.9% among the 121 patients with *TP53* gene missense mutations and only 5.6% in the 36 cases with nonsense mutations. Other types of mutations in the 55 patients (including frame shift, silent, and splice) were 3.6%. Apparently, missense mutation was most likely the cause of p53 protein mutation, leading to the high expression of p53 protein. Other mutation types were possible causes of low expression of the p53 protein. Surprisingly, in the 64 cases of *TP53*wild-type, high expression of p53 protein accounted for 35.9%, while low expression was 64.1% (41 patients, of which 26 were not expressed at all) (Figure 4(e) and Table 5).

### 3.5. A New Classification for Evaluating the Association between *TP53* Mutation/p53 Protein and Survival

To address the controversial role of *TP53* mutation in ESCC, in this study, we established a new classification for evaluating *TP53*-related survival in ESCC by combining *TP53* mutations and p53 protein expression analysis. The results clearly showed that 276 ESCC patients were divided into four groups as follows: *TP53* mutation/p53 high expression (85/276, 30.8%), *TP53* mutation/p53 low expression (127/276, 46.0%), *TP53* wild-type/p53 high expression (23/276, 8.3%), *TP53* wild-type/p53 low expression (41/276, 14.9%) (Table 5). Furthermore, the total survival time of the four groups was significantly different (*p* < 0.01), among which the *TP53* mutation/p53 high expression group had the worst survival time, followed by the *TP53* wild-type/p53 high expression group (Figure 5(e)).

### 3.6. Association between p53 Protein Expression and Clinicopathological Changes in Cohort with Large-Scale ESCC Patients

To further validate our discovery of the association between the p53 expression-related prognosis and clinicopathological changes, we assessed additional cohorts to make an analysis (Table 6). The positive expression of p53 protein was observed in 3,562 (59.1%) ESCC patients, of which 1,819 patients (51.1%) showed high expression of p53 protein. Moreover, the high expression of p53 protein in high-incidence areas was more common than in low-incidence areas with a rate and *p* value of 31.5% vs. 27.7%, *p*=0.003. High expression of p53 protein was closely correlated with poor tumor differentiation (*p* < 0.001). The frequency of high expression of p53 protein in the early ESCC (0 + I stage) was 1.27-fold higher than that in the advanced ESCC (38.2% vs. 30.0%, *p*=0.034). Furthermore, the rate of positive cancer embolus in high-expression groups was 1.31-fold higher than in low-expression groups (5.1% vs. 3.9%, *p*=0.028).

### 3.7. Independent Prognosis Marker Role of p53 Protein on Survival Analysis in 6,028 ESCC Patients

To determine whether a high expression level of the p53 protein could be used as a prognosis marker in patients with ESCC, we conducted the analysis using the p53 expression in 6,028 ESCC patients. In this cohort, the analysis showed median OS time in patients with p53 high expression and with low was 2.71 ± 0.09 and 3.08 ± 0.06 years, respectively. The presence of high expression of p53 protein was significantly associated with decreased OS (*p* < 0.001, Figure 5(f)).

### 3.8. Cox Univariate and Multivariate Regression Analyses

**Figure 6:**
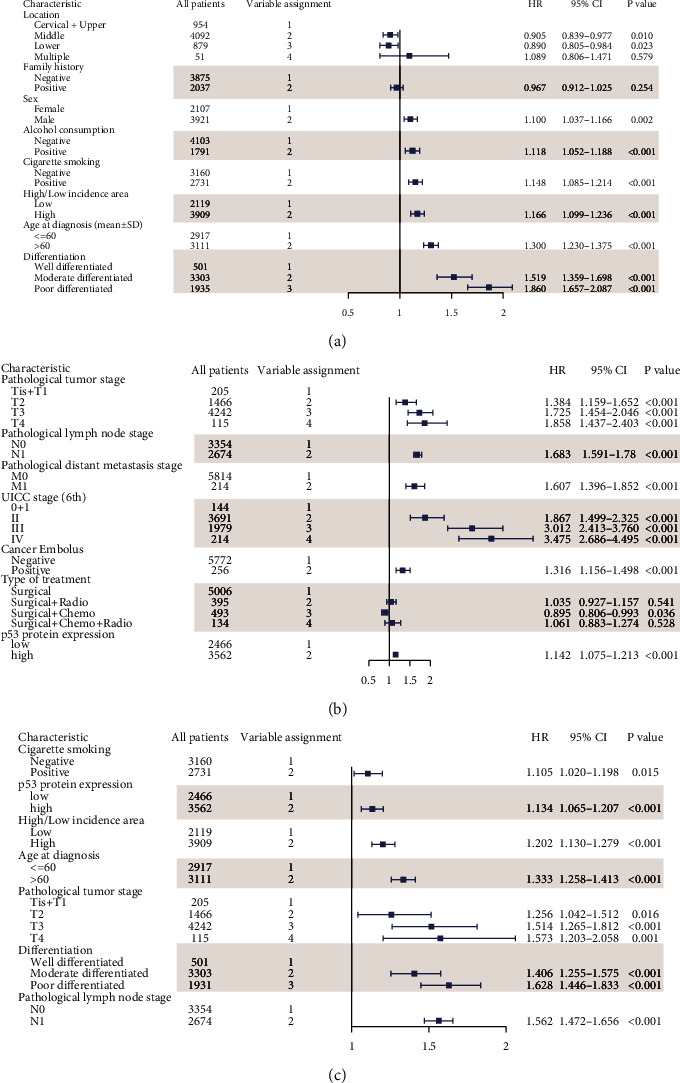
Forest plot demonstrating univariate and multivariate analysis of factors that influence OS in 6,028 ESCC patients. The results of the univariate analysis of factors that influence OS in 6,028 ESCC patients are shown in (a) and (b). The results of the multivariate analysis of factors that influence OS in 6,028 ESCC patients are shown in (c).

**Figure 7:**
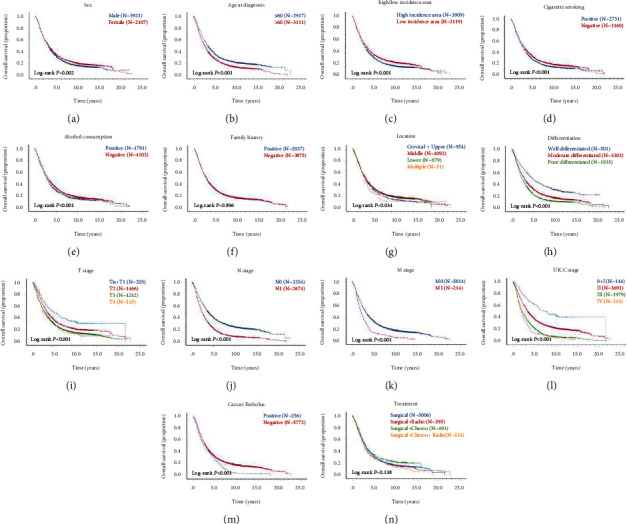
Kaplan–Meier curves analysis estimating OS following stratifying 6,028 patients with ESCC according to sex (a), age at diagnosis (b), high/low incidence area (c), cigarette smoking (d), alcohol consumption (e), family history (f), location (g), differentiation (h), T stage (i), N stage (j), M stage (k), UICC stage (l), cancer embolus (m), and treatment (n), respectively.

Cox proportional-hazard models were chosen for the univariate and multivariate analyses. The univariate Cox regression analysis demonstrated that the sex, age, high/low incidence area, cigarette smoking, alcohol consumption, location, differentiation, T stage, N stage, M stage, UICC stage, cancer embolus, and p53 protein high/low expression were dependent prognostic factors (*p* < 0.05, Figures 6(a), 6(b), and 7). In multivariate Cox regression analysis, as compared with the low expression of p53 protein, the patients with high expression (HR = 1.134, 95% CI 1.065 to 1.207) were associated with decreased survival after adjustment for the age, high/low incidence area, cigarette smoking, differentiation, and T and N stages as independent prognostic factors (*p* < 0.05, Figure 6(c)).

### 3.9. Stratified Survival Analysis by within Independent Prognostic Factors

**Figure 8:**
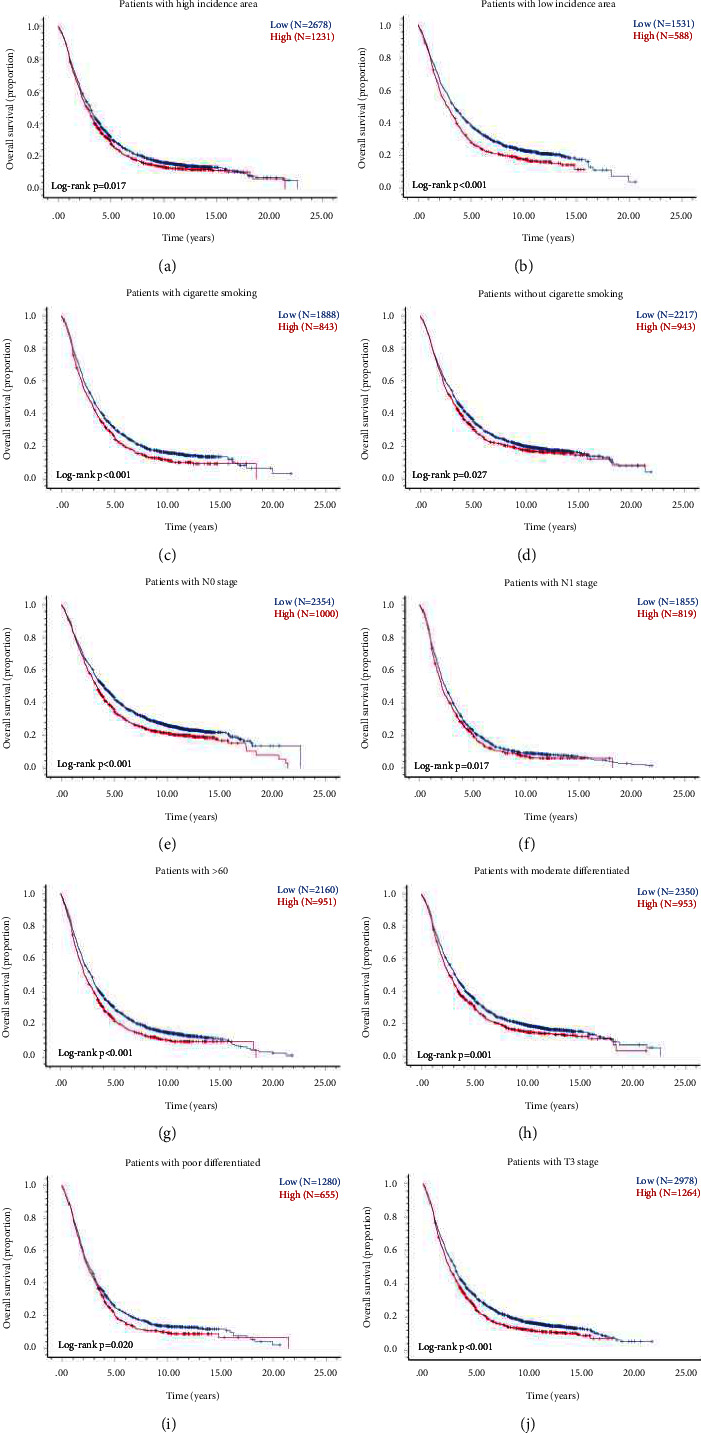
Kaplan–Meier curves survival analysis of ESCC patients with high/low p53 protein expression after stratification based on independent prognostic factors. OS of patients with p53 protein high vs. low expression in patients with high-incidence area (a) and low-incidence area (b). OS of patients with p53 protein high vs. low expression in patients with (c) and without cigarette smoking (d). OS of patients with p53 protein high vs. low expression in patients with N0 (e) and N1 stage (f). OS of patients with p53 protein high vs. low expression in patients >60 years (g). OS of patients with p53 protein high vs. low expression in patients with moderate (h) and poor differentiation (i). OS of patients with p53 protein high vs. low expression in patients with T3 stage (j).

We performed a stratified analysis to eliminate the influence of the independent prognostic factors on evaluating the expression of the p53 protein on prognosis. When patients were stratified according to high/low incidence area, cigarette smoking, and N stages, the consistent trend of OS was observed in different stratification (Figures 8(a)–8(f)). When stratified according to patients' age at diagnosis, degree of differentiation, and T stage, we only found that patients the high expression of p53 protein in >60 (*p* < 0.001, Figure 8(g)), medium and low differentiation (*p* < 0.001, Figures 8(h) and 8(i)), and T3 (*p* < 0.001, Figure 8(j)) had shorter OS than those with low expression.

## 4. Discussion

We have conducted a two-large ESCC cohort analysis to evaluate the association between *TP53* mutation statuses/the expression of the p53 protein and clinicopathological features and prognosis in ESCC patients. Most importantly, our results further confirmed that the expression of the p53 protein can reflect the prognosis of ESCC patients by using a large number of tissue samples, and high p53 expression indicates a poor prognosis. However, the mutation of the *TP53* gene has no obvious correlation with prognosis, which is controversial in previous reports.

With the rapid development of sequencing technology, the prognostic value of *TP53* has been confirmed in a variety of tumors, and the mutation of *TP53* indicates a poor prognosis across different types of human cancers. In this study, despite the high frequency of *TP53* mutations, there is no obvious evidence showing its association with clinicopathological features and prognosis in patients with ESCC. Previously, other colleagues showed that *TP53* status was correlated with tumor invasion depth, TNM stage, lymph node metastasis, distant metastasis, and differentiation degree [15]. Meanwhile, the survival time of *TP53* gene mutation and p53 protein overexpression was shorter than the control group in ESCC [15]. However, Zhao et al. showed that the high expression of p53 protein was an independent prognostic factor for survival, while the mutation of the *TP53* gene was unrelated to prognosis [26]. The conflicting results might be caused by the following reasons: of the limited number of patients, insufficient clinical follow-up, different experimental techniques, mutation sites on different target exons, and variable factors.

We further found that the frequency of the *TP53* mutation was 76.3% in ESCC, which was between 66.7% and 82.7% of the frequency of the *TP53* mutation in EC by NGS, as previously reported [27–30]. Cui et al. published WGS results for 508 cases of ESCC, which also showed a similar *TP53* mutation frequency of 74.8% [30]. The cause might be that the study population and histopathological type were different. Our study confirmed that the main mutation type of *TP53* in ESCC patients is a missense mutation, which may play a pivotal role in tumorigenesis because the p53 protein usually has positive expression, or even higher expression, owing to the accumulation of a nonfunctional protein that loss activity as a tumor suppressor, and some of which exert trans-dominant repression over the wild-type counterpart [31, 32]. *C* > *T*/*G* > *A* Transition is the predominant mutation of *TP53* gene mutations in our study, and it is a typical marker associated with betel quid chewing, tobacco use, and alcohol drinking in oral squamous cell carcinoma in Taiwanese [33]. *C* > *A*/*G* > *T* transversion is a typical feature of carcinogen exposure associated with cigarette smoking, which is the most risk factor for ESCC and lung cancer. This transversion occurred at the sites of adduct formation for the metabolites of benzo (a) pyrene, a major tobacco carcinogen (codons 157, 248, and 273) [34, 35].

To our knowledge, this is the largest sample-scale study that has examined p53 protein expression by IHC. Our study herein showed that the expression of p53 protein was associated with high/low incidence area, degree of differentiation, and cancer embolus, and revealed that high expression of p53 protein is an independent prognostic factor rather than *TP53* gene mutation, with 1.134-fold mortality risk. In previous studies, the correlation between p53 protein expression, clinicopathological features, and prognosis was significantly different [36–40]. To date, only 13 of the 30 articles have suggested that p53 protein was an adverse factor for prognosis, and the rest of the reports suggested that p53 expression had no effect on prognosis [39]. Wang et al. revealed that a more advanced TNM stage, positive lymph node metastasis, and distant metastasis were associated with p53 protein expression [41]. Although there were no significant differences between p53 expression and T stage, N stage, and M stage in our finding, our further validation of 6,028 patients demonstrated a significantly strong correlation between the expression of p53 protein and clinical phenotype and prognosis. The judgment for the p53 protein expression is different because of the variety of using antibodies and experimental methods used in IHC. In addition to the source of the tumors and the existence of tumor heterogeneity, the difference in evaluation criteria is the most critical factors leading to inconsistent conclusions in previous studies [36, 37, 40].

Our study showed that the expression of p53 protein in the high-incidence areas of EC was significantly higher than in low. The existence of high/low incidence areas of EC is one of the prominent epidemiological features of ESCC, suggesting that environmental factors play an important role in the pathogenesis of ESCC. In high-incidence areas of EC, exposure to environmental carcinogens (such as nitrite and mold-contaminated foods) may lead to carcinogenesis of the esophageal squamous epithelium. *TP53* is one of the most susceptible genes to environmental carcinogens in the process of tumor induction. Aflatoxin B1 (AFB1) are potent carcinogens, which induces an arginine to serine at a single base substitution at the third base of codon 249 in *TP53* (*G* > *T* transversion) [42, 43]. N-nitroso compounds (NOC) are an alkylating agent and cause guanine alkylation to generate O^6^-alkylguanine, which results in *C* > *T*/*G* > *A* transition during DNA replication when paired with thymine [44–46]. The DNA repair protein O^6^-alkylguanine-DNA alkyltransferase (AGT) specifically repairs O^6^-alkylguanine adducts in DNA. However, AGT mutations occurred more frequently in patients in high-incidence areas of esophageal cancer than in the normal population [47].

In ESCC, it is not feasible to determine the presence of *TP53* gene mutations through the loss of p53 protein expression. When the *TP53* gene is mutated, it will cause different changes in proteins. Nonsense, frameshift, silent, and splice mutations usually cause protein truncation and make the synthesis of normal p53 protein interfere in order to cause LOF, and p53 protein is not expressed either [21]. The situation became complicated when a missense mutation occurred because the p53 protein can be caused by various missense mutations. Some will still have the function of wild-type p53 protein, and some will only retain part of the function and acquire gain GOF, which is associated with malignancy, invasion, and metastasis [48]. Due to the broad existence of LOF and GOF of the *TP53* mutation, false-positive or false-negative levels of p53 protein will be detected by using IHC in clinical applications, which might not be a reliable method for evaluating the function of the *TP53* mutation [49]. Our observation confirmed that immunohistochemical detection of p53 protein expression could more effectively reflect the relationship with prognosis and clinicopathologic features than the mutation status of the *TP53* gene in ESCC, which was further supported by studies with a larger sample.

The limitation of this study is the representativeness and accuracy of the application of TMA-based IHC showing the expression of p53 protein with extreme heterogeneity. To conclude, laboratory techniques need to be used to detect p53 protein in TMAs, and the criterion of IHC evaluation must be constant. Limited tissue sampling cannot effectively reflect the real status of the whole section because of tumor heterogeneity. Our research simultaneously detected p53 protein in a part of the original pathological sections made TMAs to reduce potential limits, and used the same scoring criteria for judgment. The results are no different from those in TMAs.

In conclusion, *TP53* is the most mutated gene in ESCC, showing great potential to be a diagnostic biomarker in treating cancer. However, the role of *TP53* mutation and expression of p53 in ESCC still remains unclear. Our study, involving two large-scale cohorts, was the first to conduct genomic profiling and expression of the p53 protein, while demonstrating the association with clinical phenotypes and prognosis. Our findings show that the expression of p53 protein is more effective in predicting clinicopathological features and prognosis, is a valid biomarker of an unfavorable prognosis, and will contribute to clinical diagnosis, prognosis prediction, and targeted therapy.

## Figures and Tables

**Table 1:** Baseline characteristics of patients with ESCC.

Characteristics	The patients undergoing *TP53* mutation analysis,		The patients with p53 IHC staining in TMA,	
	*n* = 316	(%)	*n* = 6028	(%)
Sex				
Female	117	37.0	2107	35.0
Male	199	63.0	3921	65.0

Age at diagnosis (mean ± SD)	61.39 ± 8.18		60.92 ± 8.28	
≤60	136	43.0	2917	48.4
>60	180	57.0	3111	51.6

High/low incidence area^★^				
Low	64	20.3	2119	35.2
High	252	79.7	3909	64.8

Cigarette smoking				
Negative	178	56.3	3160	52.4
Positive	136	43.1	2731	45.3
Missing	2	0.6	137	2.3

Alcohol consumption				
Negative	184	58.2	4103	68.1
Positive	130	41.1	1791	29.7
Missing	2	0.6	134	2.2

Family history				
Positive	131	41.5	2037	33.8
Negative	174	55.0	3875	64.3
Missing	11	3.5	116	1.9

Location				
Cervical + upper	45	14.2	954	15.8
Middle	182	57.6	4092	67.9
Lower	68	21.5	879	14.6
Multiple	15	4.7	51	0.8
No record	6	1.9	52	0.9

Differentiation				
Well differentiated	25	7.9	501	8.3
Moderate differentiated	195	61.7	3303	54.8
Poor differentiated	91	28.8	1935	32.1
No record	5	1.6	289	4.8

Pathological T stage				
Tis + T1	18	5.7	205	3.4
T2	83	26.3	1466	24.3
T3	203	64.2	4242	70.4
T4	12	3.8	115	1.9

Pathological N stage				
N0	174	55.1	3354	55.6
N1	142	44.9	2674	44.4

Pathological M stage				
M0	308	97.5	5814	96.4
M1	8	2.5	214	3.6

UICC stage (6th)				
0 + I	11	3.8	144	2.4
II	188	59.5	3691	61.2
III	109	34.2	1979	32.8
IV	8	2.5	214	3.6

Cancer embolus				
Positive	21	6.6	256	4.2
Negative	295	93.4	5772	95.8

Type of treatment				
Surgical	293	92.7	5006	83.0
Surgical + chemo	11	3.5	493	8.2
Surgical + radio	8	2.5	395	6.6
Surgical + chemo + radio	4	1.3	134	2.2

^★^areas with esophageal cancer incidence ≥50/100,000 are high-incidence area for esophageal cancer; on the contrary, areas with esophageal cancer incidence <50/100,000 is low-incidence area for esophageal cancer.

**Table 2:** Comparison of clinicopathologic features between mutation and wild-type*TP53* in patients with ESCC.

Characteristics	All patients,	Wild-type*TP53*,		Mutation *TP53*,		*p* value
	*n* = 316	*n* = 75	(%)	*n* = 241	(%)	
Sex						0.736
Female	117	29	24.8	88	75.2	
Male	199	46	23.1	153	76.9	
Age at diagnosis (mean ± SD)						0.543
≤60	136	30	22.1	106	77.9	
>60	180	45	25.0	135	75.0	
High/low incidence area						0.551
Low	64	17	26.6	47	73.4	
High	252	58	23.0	194	77.0	
Cigarette smoking						0.277
Negative	178	46	25.8	132	74.2	
Positive	136	28	20.6	108	79.4	
Alcohol consumption						0.211
Negative	184	48	26.1	136	73.9	
Positive	130	26	20.0	104	80.0	
Family history						0.984
Negative	174	41	23.6	133	76.4	
Positive	131	31	23.7	100	76.3	
Location						0.904^*∗*^
Cervical + upper	45	12	26.7	33	73.3	
Middle	182	42	23.1	140	76.9	
Lower	68	15	22.1	53	77.9	
Multiple	15	4	26.7	11	73.3	
Differentiation						0.260
Well differentiated	25	3	12.0	22	88.0	
Moderate differentiated	195	50	25.6	145	74.4	
Poor differentiated	91	19	20.9	72	79.1	
Pathological T stage						0.239^*∗*^
Tis + T1	18	7	38.9	11	61.1	
T2	83	17	20.5	66	79.5	
T3 + T4	215	51	23.7	164	76.3	
Pathological N stage						0.130
N0	174	47	27.0	127	73.0	
N1	142	28	19.7	114	80.3	
Pathological M stage						1.000^*∗*^
M0	308	73	23.7	235	76.3	
M1	8	2	25.0	6	75.0	
UICC stage (6th)						0.053^*∗*^
0 + I	11	5	45.5	6	54.5	
II	188	49	26.1	139	73.9	
III + IV	117	21	17.9	96	82.1	
Cancer embolus						0.590^*∗*^
Negative	295	69	23.4	226	76.6	
Positive	21	6	28.6	15	71.4	
Type of treatment						0.211
Surgical	293	72	24.6	221	75.4	
Surgical + chemo/radio	23	3	13.0	20	87.0	

^*∗*^the differences among categoric variables were analyzed using the Fisher exact test.

**Table 3:** Characteristics of the ESCC patients undergoing *TP53* mutation detection.

No	Patient ID	Sex	Age	UICC stage	Sequence source	Mutation status	Number of mutations	p53 IHC (positive/negative)	p53 IHC (low/high)	Survival status	Survival time (years)
1	CE12T	M	72	IVB	WES	*TP53* wild-type	0	Positive	Low	Dead	1.70
2	CE18T	M	50	III	WES	*TP53* mutation	1	Negative	Low	Dead	4.73
3	CE16T	M	69	IIA	WES	*TP53* wild-type	0	Positive	Low	Dead	1.81
4	CE4T	M	64	IIB	WES	*TP53* mutation	1	Negative	Low	Dead	3.95
5	CE7T	M	70	III	WES	*TP53* mutation	1	Positive	Low	Dead	5.00
6	CE15T	M	57	III	WES	*TP53* mutation	1	Positive	Low	Dead	5.81
7	CE30T	M	56	IIA	WES	*TP53* wild-type	0	Negative	Low	Alive	10.51
8	CE20T	M	72	IIA	WES	*TP53* mutation	1	Positive	Low	Dead	4.07
9	CE26T	M	71	III	WES	*TP53* mutation	2	Positive	High	Dead	6.16
10	CE5T	F	75	III	WES	*TP53* mutation	1	Positive	Low	Dead	5.11
11	CE25T	M	64	IIA	WES	*TP53* wild-type	0	Positive	High	Dead	2.05
12	CE24T	M	58	IIA	WES	*TP53* mutation	1	Positive	High	Alive	11.04
13	CE21T	M	54	IIA	WES	*TP53* mutation	2	Negative	Low	Dead	6.50
14	CE27T	M	64	III	WES	*TP53* mutation	1	Positive	High	Dead	2.41
15	EC888T	M	59	III	TRS	*TP53* wild-type	0	Positive	High	Alive	5.05
16	EC889T	M	62	III	TRS	*TP53* mutation	1	Positive	High	Dead	0.79
17	EC891T	M	55	IIB	TRS	*TP53* wild-type	0	Negative	Low	Dead	1.75
18	EC892T	M	60	III	TRS	*TP53* mutation	1	Positive	High	Dead	3.15
19	EC893T	F	64	IIA	TRS	*TP53* mutation	1	Positive	High	Alive	4.71
20	EC1038T	M	74	IV	TRS	*TP53* mutation	1	Positive	Low	Dead	1.49
21	EC1035T	M	65	III	TRS	*TP53* wild-type	0	Positive	High	Dead	2.27
22	EC1032T	F	51	IIA	TRS	*TP53* mutation	1	Positive	High	Dead	2.27
23	EC632T	M	59	IIA	TRS	*TP53* mutation	1	Positive	High	Alive	4.78
24	EC636T	M	64	IIB	TRS	*TP53* mutation	1	Negative	Low	Alive	4.77
25	EC090T	M	47	III	WES	*TP53* mutation	1	Positive	High	Alive	1.41
26	EC633T	F	63	I	TRS	*TP53* mutation	1	Positive	Low	Alive	4.80
27	EC640T	M	58	IIA	TRS	*TP53* mutation	1	Negative	Low	Alive	4.81
28	EC643T	F	67	IIA	TRS	*TP53* mutation	1	Positive	Low	Dead	2.54
29	EC644T	M	61	III	TRS	*TP53* mutation	1	Negative	Low	Dead	2.33
30	EC641T	F	60	IIA	TRS	*TP53* mutation	1	Negative	Low	Alive	4.84
31	EC967T	M	69	IIA	TRS	*TP53* mutation	2	Negative	Low	Alive	4.84
32	EC968T	F	63	IIA	TRS	*TP53* mutation	1	Positive	Low	Alive	4.85
33	EC966T	M	69	IIA	TRS	*TP53* mutation	2	Positive	High	Dead	2.37
34	EC965T	F	71	IIA	TRS	*TP53* mutation	1	Positive	High	Dead	0.95
35	EC1024T	F	65	IVA	TRS	*TP53* mutation	1	Positive	Low	Dead	4.03
36	EC190T	M	50	IIA	WES	*TP53* mutation	2	Positive	Low	Alive	10.80
37	EC969T	M	63	III	TRS	*TP53* mutation	1	Positive	High	Alive	4.88
38	EC970T	M	66	III	TRS	*TP53* wild-type	0	Negative	Low	Dead	0.13
39	EC971T	M	66	III	TRS	*TP53* mutation	1	Negative	Low	Alive	5.21
40	EC973T	F	63	IIB	TRS	*TP53* mutation	1	Negative	Low	Dead	0.88
41	EC975T	M	62	III	TRS	*TP53* mutation	1	Positive	Low	Alive	4.88
42	EC976T	F	79	IIA	TRS	*TP53* mutation	1	Positive	Low	Dead	1.89
43	EC978T	M	57	IIA	TRS	*TP53* mutation	1	Negative	Low	Alive	5.26
44	EC981T	M	69	III	TRS	*TP53* mutation	1	Negative	Low	Alive	4.90
45	EC982T	M	64	IIB	TRS	*TP53* mutation	1	Positive	Low	Alive	4.90
46	EC983T	M	44	IIA	TRS	*TP53* mutation	1	Negative	Low	Alive	3.95
47	EC191T	M	59	III	WES	*TP53* mutation	1	Positive	Low	Dead	4.18
48	EC984T	M	63	III	TRS	*TP53* mutation	1	Positive	Low	Dead	1.36
49	EC985T	M	51	III	TRS	*TP53* mutation	1	Negative	Low	Alive	5.30
50	EC986T	M	72	IIA	TRS	*TP53* mutation	1	Negative	Low	Dead	3.24
51	EC987T	M	67	III	TRS	*TP53* mutation	1	Positive	Low	Alive	4.94
52	EC990T	M	73	III	TRS	*TP53* mutation	1	Negative	Low	Dead	1.64
53	EC991T	M	67	IVA	TRS	*TP53* mutation	1	Positive	Low	Dead	2.42
54	EC993T	M	56	III	TRS	*TP53* wild-type	0	Negative	Low	Alive	3.12
55	EC992T	M	59	IIA	TRS	*TP53* mutation	1	Positive	Low	Alive	4.96
56	EC994T	M	47	III	TRS	*TP53* mutation	1	Positive	Low	Alive	2.81
57	EC995T	M	68	IIA	TRS	*TP53* wild-type	0	Negative	Low	Alive	4.98
58	EC088T	M	58	III	WES	*TP53* mutation	1	Negative	Low	Dead	6.07
59	EC996T	F	74	IIA	TRS	*TP53* mutation	1	Positive	High	Alive	4.98
60	EC630T	F	57	IIA	TRS	*TP53* mutation	1	Positive	High	Dead	2.79
61	EC997T	F	61	IIA	TRS	*TP53* mutation	1	Positive	Low	Alive	4.98
62	EC629T	M	67	IIB	TRS	*TP53* mutation	1	Positive	Low	Dead	2.43
63	EC1006T	M	58	III	TRS	*TP53* mutation	2	Positive	High	Alive	5.04
64	EC885T	M	65	IIB	TRS	*TP53* mutation	2	Positive	Low	Dead	4.01
65	EC1005T	F	73	III	TRS	*TP53* mutation	1	Positive	Low	Dead	2.92
66	EC886T	M	62	IIB	TRS	*TP53* wild-type	0	Positive	High	Dead	1.56
67	G008_T	M	71	IIA	WES	*TP53* mutation	1	Positive	High	Dead	2.53
68	NR131225_2T	F	70	III	WGS	*TP53* mutation	1	Negative	Low	Alive	5.29
69	EC080T	M	70	I	WES	*TP53* mutation	1	Positive	Low	Dead	2.70
70	NR131225_3T	M	55	IIA	WGS	*TP53* mutation	1	Positive	High	Alive	5.32
71	NR131225_5T	F	67	IIA	WGS	*TP53* mutation	1	Negative	Low	Dead	0.93
72	NR131225_9T	M	67	IIA	WGS	*TP53* mutation	1	Negative	Low	Alive	5.67
73	NR131225_4T	M	74	IIA	WGS	*TP53* mutation	1	Positive	Low	Dead	3.61
74	NR131225_10T	M	77	III	WGS	*TP53* wild-type	0	Negative	Low	Dead	2.50
75	NR131225_1T	F	57	III	WGS	*TP53* mutation	1	Positive	High	Dead	3.70
76	NR131225_8T	M	58	III	WGS	*TP53* wild-type	0	Positive	Low	Alive	5.36
77	NR131225_6T	M	60	IIA	WGS	*TP53* mutation	1	Positive	High	Dead	2.83
78	NR131121_10T	M	53	IIA	WGS	*TP53* mutation	1	Positive	High	Dead	0.66
79	NR131121_9T	M	66	IIB	WGS	*TP53* mutation	1	Positive	High	Dead	1.00
80	EC076T	M	60	III	WES	*TP53* wild-type	0	Positive	Low	Alive	11.19
81	NR131121_8T	M	60	IIA	WGS	*TP53* mutation	1	Positive	Low	Dead	4.00
82	NR131121_7T	M	59	IIB	WGS	*TP53* mutation	1	Positive	Low	Dead	5.27
83	NR131121_6T	M	69	IIA	WGS	*TP53* mutation	1	Positive	High	Dead	1.01
84	NR131121_5T	F	64	III	WGS	*TP53* mutation	1	Positive	Low	Alive	4.80
85	NR131121_4T	M	65	III	WGS	*TP53* mutation	1	Positive	High	Dead	0.58
86	NR131121_3T	F	72	IIA	WGS	*TP53* mutation	1	Positive	Low	Alive	5.57
87	NR131121_2T	M	61	IIA	WGS	*TP53* mutation	2	Positive	High	Dead	4.49
88	NR131121_1T	M	63	IIA	WGS	*TP53* mutation	1	Positive	High	Dead	2.03
89	TW2352AF	M	55	IIA	WES	*TP53* mutation	1	Positive	Low	Alive	7.87
90	TW2351BF	M	46	IIA	WES	*TP53* mutation	1	Positive	High	Alive	8.28
91	EC062T	F	59	III	WES	*TP53* mutation	1	Positive	Low	Dead	6.49
92	EC129T	M	54	IIA	WES	*TP53* mutation	1	Negative	Low	Alive	10.30
93	EC127T	F	72	IIB	WES	*TP53* mutation	1	Negative	Low	Dead	8.45
94	MC16T	M	62	IIA	WES	*TP53* mutation	1	Negative	Low	Dead	7.80
95	MC17T	M	50	IIA	WES	*TP53* mutation	2	Positive	Low	Dead	6.87
96	MC13T	M	54	III	WES	*TP53* mutation	1	Positive	High	Dead	4.25
97	MC7T	M	53	III	WES	*TP53* mutation	2	Positive	High	Dead	0.75
98	TW258AF	M	40	III	WES	*TP53* mutation	1	Positive	High	Dead	0.60
99	FE0008T	M	64	III	TRS	*TP53* mutation	1	Positive	High	Dead	0.83
100	EC123T	M	56	IIA	WES	*TP53* mutation	1	Positive	Low	Dead	4.06
101	G556T	M	64	III	TRS	*TP53* mutation	1	Positive	Low	Alive	2.65
102	G406T	M	69	III	TRS	*TP53* mutation	1	Positive	High	Dead	0.46
103	FG0067T	M	68	III	TRS	*TP53* mutation	2	Positive	High	Alive	1.10
104	EC745T	M	64	IIA	TRS	*TP53* wild-type	0	Negative	Low	Alive	2.95
105	G414T	M	57	III	TRS	*TP53* mutation	1	Negative	Low	Alive	3.11
106	EC727T	M	70	IIA	TRS	*TP53* mutation	1	Positive	High	Alive	3.14
107	EC720T	F	74	IIA	TRS	*TP53* mutation	1	Negative	Low	Dead	1.16
108	EC718T	M	64	III	TRS	*TP53* wild-type	0	Negative	Low	Dead	1.66
109	EC721T	M	56	III	TRS	*TP53* mutation	1	Positive	High	Dead	0.48
110	EC943T	M	65	IIA	TRS	*TP53* mutation	1	Positive	High	Alive	3.79
111	EC121T	M	59	III	WES	*TP53* mutation	1	Positive	High	Dead	1.00
112	EC854T	F	64	IIA	TRS	*TP53* mutation	2	Positive	High	Alive	3.44
113	EC868T	F	52	III	TRS	*TP53* mutation	1	Positive	Low	Alive	3.44
114	EC686T	F	59	IIA	TRS	*TP53* mutation	2	Positive	Low	Alive	3.43
115	EC687T	F	58	IIA	TRS	*TP53* wild-type	0	Positive	High	Dead	0.92
116	EC685T	F	78	IIA	TRS	*TP53* wild-type	0	Positive	Low	Alive	3.47
117	EC884T	M	63	IIA	TRS	*TP53* wild-type	0	Negative	Low	Alive	3.45
118	EC883T	M	62	III	TRS	*TP53* mutation	2	Positive	High	Alive	3.49
119	EC881T	F	62	III	TRS	*TP53* mutation	1	Positive	High	Dead	1.24
120	EC877T	M	68	III	TRS	*TP53* mutation	1	Positive	High	Alive	3.51
121	EC880T	M	68	IIA	TRS	*TP53* mutation	1	Positive	High	Alive	3.47
122	EC122T	M	52	III	WES	*TP53* mutation	1	Negative	Low	Alive	11.47
123	EC876T	F	47	IIA	TRS	*TP53* wild-type	0	Negative	Low	Alive	3.51
124	EC871T	M	78	IIA	TRS	*TP53* mutation	1	Positive	High	Alive	3.51
125	EC874T	F	64	IIA	TRS	*TP53* mutation	1	Negative	Low	Alive	3.53
126	EC855T	F	67	I	TRS	*TP53* wild-type	0	Positive	High	Dead	1.00
127	EC870T	F	65	III	TRS	*TP53* wild-type	0	Positive	High	Alive	3.91
128	EC872T	M	64	III	TRS	*TP53* mutation	1	Positive	High	Dead	0.89
129	EC875T	M	68	IIA	TRS	*TP53* mutation	1	Positive	Low	Dead	1.99
130	EC869T	M	64	I	TRS	*TP53* mutation	1	Positive	High	Dead	1.53
131	EC811T	F	65	IIA	TRS	*TP53* wild-type	0	Positive	High	Dead	2.22
132	G479T	M	77	IV	TRS	*TP53* mutation	1	Positive	Low	Dead	0.57
133	EC124T	M	62	III	WES	*TP53* mutation	1	Positive	High	Dead	2.07
134	EC814T	F	61	IIA	TRS	*TP53* mutation	1	Negative	Low	Alive	3.58
135	EC813T	F	60	IIA	TRS	*TP53* mutation	1	Positive	High	Alive	3.58
136	EC816T	F	66	IIA	TRS	*TP53* wild-type	0	Negative	Low	Dead	3.21
137	EC812T	F	72	IIA	TRS	*TP53* wild-type	0	Positive	High	Dead	0.64
138	EC822T	M	63	IIA	TRS	*TP53* mutation	1	Positive	Low	Alive	3.95
139	EC818T	F	53	IIA	TRS	*TP53* mutation	2	Positive	Low	Alive	3.58
140	EC821T	F	64	IIA	TRS	*TP53* mutation	1	Positive	High	Alive	3.59
141	EC853T	F	65	IIA	TRS	*TP53* wild-type	0	Positive	High	Alive	2.64
142	EC832T	F	72	IIA	TRS	*TP53* mutation	1	Negative	Low	Dead	0.25
143	EC851T	F	67	IIA	TRS	*TP53* mutation	1	Negative	Low	Alive	3.64
144	EC120T	M	65	IIB	WES	*TP53* mutation	1	Positive	High	Dead	3.49
145	EC835T	M	63	IIA	TRS	*TP53* mutation	1	Negative	Low	Dead	1.26
146	EC834T	F	59	IIA	TRS	*TP53* mutation	1	Negative	Low	Alive	3.66
147	EC859T	M	61	III	TRS	*TP53* mutation	1	Negative	Low	Dead	1.02
148	EC850T	M	72	IIA	TRS	*TP53* wild-type	0	Positive	Low	Alive	3.69
149	EC838T	M	58	IIB	TRS	*TP53* mutation	1	Positive	High	Alive	2.67
150	EC849T	M	64	IIA	TRS	*TP53* mutation	1	Positive	High	Dead	1.26
151	EC848T	M	53	III	TRS	*TP53* mutation	1	Positive	High	Alive	3.73
152	EC847T	F	65	IIA	TRS	*TP53* mutation	1	Negative	Low	Alive	3.72
153	EC845T	M	57	I	TRS	*TP53* mutation	1	Negative	Low	Alive	3.75
154	EC932T	F	60	IIA	TRS	*TP53* mutation	1	Negative	Low	Dead	2.10
155	EC116T	F	58	IIA	WES	*TP53* mutation	1	Positive	High	Alive	11.53
156	EC934T	M	70	IIA	TRS	*TP53* wild-type	0	Negative	Low	Dead	1.44
157	EC935T	F	64	I	TRS	*TP53* wild-type	0	Negative	Low	Alive	3.76
158	EC933T	F	66	IIA	TRS	*TP53* mutation	1	Negative	Low	Dead	3.20
159	EC941T	M	62	III	TRS	*TP53* mutation	1	Negative	Low	Dead	2.57
160	EC942T	F	73	IIA	TRS	*TP53* mutation	1	Negative	Low	Alive	3.78
161	EC945T	M	66	IIA	TRS	*TP53* mutation	1	Positive	High	Alive	3.80
162	EC944T	F	65	IIB	TRS	*TP53* mutation	1	Positive	High	Dead	1.31
163	EC936T	F	58	IIA	TRS	*TP53* wild-type	0	Positive	High	Alive	3.79
164	EC937T	F	60	IIB	TRS	*TP53* wild-type	0	Positive	High	Alive	3.79
165	EC948T	F	60	IIB	TRS	*TP53* mutation	1	Negative	Low	Alive	3.81
166	EC117T	M	50	III	WES	*TP53* mutation	3	Positive	High	Dead	1.03
167	EC146T	F	51	III	WES	*TP53* mutation	1	Positive	Low	Dead	3.25
168	EC940T	F	69	IIA	TRS	*TP53* mutation	1	Negative	Low	Dead	3.09
169	EC938T	F	63	IIA	TRS	*TP53* wild-type	0	Positive	Low	Dead	1.08
170	EC939T	F	52	IIA	TRS	*TP53* wild-type	0	Positive	High	Dead	0.92
171	EC951T	F	63	IIA	TRS	*TP53* mutation	2	Positive	High	Alive	3.85
172	EC952T	F	61	IIA	TRS	*TP53* mutation	1	Negative	Low	Alive	3.85
173	EC963T	F	56	IIA	TRS	*TP53* mutation	2	Negative	Low	Alive	3.86
174	EC958T	F	71	III	TRS	*TP53* mutation	1	Negative	Low	Alive	3.86
175	EC959T	F	57	III	TRS	*TP53* wild-type	0	Negative	Low	Dead	1.32
176	EC961T	F	48	IIA	TRS	*TP53* mutation	2	Positive	High	Alive	3.88
177	EC962T	M	52	IIA	TRS	*TP53* mutation	1	Positive	Low	Alive	4.24
178	EC118T	F	65	IIB	WES	*TP53* mutation	1	Negative	Low	Dead	3.05
179	EC956T	M	55	IIA	TRS	*TP53* wild-type	0	Positive	Low	Alive	3.91
180	EC960T	M	51	III	TRS	*TP53* mutation	2	Positive	High	Dead	1.98
181	EC954T	F	69	IIA	TRS	*TP53* wild-type	0	Positive	Low	Alive	3.91
182	EC955T	F	52	IIB	TRS	*TP53* mutation	1	Negative	Low	Dead	1.20
183	EC953T	M	61	IIA	TRS	*TP53* wild-type	0	Positive	High	Dead	1.27
184	EC802T	F	63	III	TRS	*TP53* mutation	1	Negative	Low	Dead	1.48
185	EC793T	M	69	IIA	TRS	*TP53* mutation	1	Negative	Low	Alive	3.95
186	EC789T	M	63	IIA	TRS	*TP53* mutation	1	Negative	Low	Alive	3.95
187	EC797T	M	58	III	TRS	*TP53* mutation	1	Negative	Low	Alive	4.33
188	EC798T	F	59	IIA	TRS	*TP53* mutation	1	Negative	Low	Alive	3.96
189	EC115T	F	69	IIA	WES	*TP53* mutation	2	Positive	High	Dead	1.48
190	EC788T	F	58	IIA	TRS	*TP53* wild-type	0	Positive	Low	Dead	2.98
191	EC792T	M	70	IIA	TRS	*TP53* mutation	1	Positive	Low	Dead	3.21
192	EC791T	M	61	III	TRS	*TP53* mutation	3	Positive	High	Dead	1.25
193	EC790T	M	79	III	TRS	*TP53* mutation	1	Negative	Low	Dead	1.78
194	EC794T	M	65	III	TRS	*TP53* mutation	2	Positive	High	Dead	1.74
195	EC783T	M	75	III	TRS	*TP53* mutation	1	Positive	High	Alive	3.97
196	EC782T	M	67	IIA	TRS	*TP53* wild-type	0	Positive	Low	Dead	2.42
197	EC795T	F	69	IIA	TRS	*TP53* mutation	1	Positive	High	Dead	1.51
198	EC787T	M	59	III	TRS	*TP53* mutation	1	Positive	High	Dead	0.84
199	G521T	F	65	IIA	TRS	*TP53* mutation	1	Negative	Low	Alive	3.63
200	EC113T	M	64	III	WES	*TP53* mutation	2	Positive	Low	Alive	11.54
201	EC780T	M	49	IIA	TRS	*TP53* mutation	1	Negative	Low	Dead	1.96
202	EC786T	M	66	IIA	TRS	*TP53* mutation	1	Positive	High	Alive	4.05
203	EC781T	M	72	IIA	TRS	*TP53* mutation	1	Positive	High	Dead	1.33
204	EC785T	M	47	IIA	TRS	*TP53* wild-type	0	Negative	Low	Alive	4.06
205	EC779T	F	52	III	TRS	*TP53* mutation	1	Positive	High	Alive	4.42
206	EC766T	F	62	III	TRS	*TP53* mutation	1	Positive	Low	Dead	1.61
207	EC769T	F	68	IIA	TRS	*TP53* mutation	1	Positive	High	Alive	4.46
208	EC807T	F	82	IIA	TRS	*TP53* mutation	1	Positive	High	Alive	4.11
209	EC900T	F	64	IIA	TRS	*TP53* mutation	1	Negative	Low	Alive	4.11
210	EC898T	M	59	III	TRS	*TP53* mutation	1	Negative	Low	Alive	4.13
211	EC015T	M	63	III	WES	*TP53* wild-type	0	Positive	Low	Dead	2.25
212	EC904T	F	48	IIA	TRS	*TP53* mutation	1	Positive	High	Alive	4.12
213	EC899T	M	60	IIB	TRS	*TP53* wild-type	0	Positive	High	Alive	4.12
214	EC908T	F	56	III	TRS	*TP53* mutation	1	Positive	High	Dead	1.88
215	EC907T	M	60	IIA	TRS	*TP53* mutation	1	Positive	High	Dead	15.80
216	EC901T	F	76	III	TRS	*TP53* wild-type	0	Positive	Low	Dead	3.13
217	EC902T	M	48	III	TRS	*TP53* mutation	1	Positive	Low	Alive	4.15
218	EC906T	M	49	IIA	TRS	*TP53* mutation	1	Not detected	Not detected	Alive	4.51
219	EC972T	M	63	IIA	TRS	*TP53* wild-type	0	Positive	High	Dead	0.98
220	EC910T	M	65	IIA	TRS	*TP53* wild-type	0	Negative	Low	Alive	4.14
221	G048_T	F	64	III	WES	*TP53* wild-type	0	Positive	High	Dead	0.70
222	TW094BF	M	40	III	WES	*TP53* mutation	2	Positive	Low	Dead	2.74
223	G042_T	M	65	III	WES	*TP53* mutation	1	Negative	Low	Alive	4.16
224	EC912T	F	63	IIA	TRS	*TP53* wild-type	0	Positive	High	Dead	2.14
225	EC911T	F	52	IIA	TRS	*TP53* wild-type	0	Negative	Low	Dead	0.46
226	EC909T	F	56	IIB	TRS	*TP53* mutation	1	Negative	Low	Alive	3.12
227	EC916T	F	62	IIA	TRS	*TP53* mutation	1	Positive	Low	Alive	4.18
228	EC915T	M	67	IIA	TRS	*TP53* wild-type	0	Negative	Low	Alive	4.19
229	EC913T	M	70	IIA	TRS	*TP53* mutation	1	Negative	Low	Dead	3.05
230	EC917T	F	62	IIA	TRS	*TP53* wild-type	0	Positive	High	Dead	3.65
231	EC920T	F	56	III	TRS	*TP53* mutation	1	Positive	High	Dead	2.15
232	EC918T	M	68	III	TRS	*TP53* mutation	1	Positive	High	Dead	2.21
233	TW890BF	M	65	III	WES	*TP53* mutation	1	Positive	Low	Dead	2.35
234	G039_T	M	64	IIA	WES	*TP53* wild-type	0	Negative	Low	Alive	4.24
235	EC922T	M	74	IIB	TRS	*TP53* wild-type	0	Positive	Low	Dead	3.49
236	EC921T	F	60	IIB	TRS	*TP53* mutation	1	Negative	Low	Dead	2.00
237	EC923T	M	68	III	TRS	*TP53* wild-type	0	Negative	Low	Dead	2.90
238	EC924T	M	57	IIA	TRS	*TP53* mutation	1	Positive	High	Dead	3.30
239	EC683T	M	46	IIA	TRS	*TP53* wild-type	0	Negative	Low	Alive	4.42
240	EC684T	M	56	III	TRS	*TP53* mutation	1	Negative	Low	Dead	3.84
241	EC682T	M	63	IIA	TRS	*TP53* mutation	1	Negative	Low	Alive	4.42
242	EC679T	M	59	III	TRS	*TP53* wild-type	0	Negative	Low	Dead	2.31
243	EC680T	F	62	IIA	TRS	*TP53* mutation	1	Negative	Low	Alive	4.42
244	EC055T	M	45	III	WES	*TP53* mutation	1	Positive	High	Dead	0.88
245	EC681T	M	70	IIA	TRS	*TP53* mutation	1	Negative	Low	Alive	4.44
246	EC678T	F	48	IIB	TRS	*TP53* wild-type	0	Positive	Low	Dead	1.37
247	EC676T	M	57	III	TRS	*TP53* mutation	1	Negative	Low	Dead	0.98
248	EC677T	M	46	III	TRS	*TP53* mutation	1	Negative	Low	Alive	4.44
249	EC672T	M	57	III	TRS	*TP53* mutation	1	Negative	Low	Dead	1.83
250	EC666T	F	67	IVA	TRS	*TP53* mutation	1	Negative	Low	Dead	1.54
251	EC660T	F	66	IIA	TRS	*TP53* mutation	1	Positive	High	Alive	4.50
252	EC658T	M	59	I	TRS	*TP53* wild-type	0	Positive	High	Alive	4.52
253	EC659T	F	63	III	TRS	*TP53* mutation	1	Positive	High	Alive	4.87
254	EC1002T	F	62	III	TRS	*TP53* mutation	1	Positive	High	Alive	4.65
255	EC039T	M	74	IIA	WES	*TP53* wild-type	0	Negative	Low	Dead	0.35
256	EC656T	F	72	IIA	TRS	*TP53* wild-type	0	Negative	Low	Alive	4.51
257	EC654T	F	61	III	TRS	*TP53* wild-type	0	Positive	High	Dead	0.96
258	EC651T	M	51	IIB	TRS	*TP53* mutation	1	Positive	High	Alive	4.53
259	EC645T	F	57	IIA	TRS	*TP53* mutation	1	Negative	Low	Alive	4.55
260	EC928T	F	61	IIB	TRS	*TP53* mutation	2	Positive	Low	Alive	4.58
261	EC926T	M	73	IIA	TRS	*TP53* mutation	1	Negative	Low	Alive	4.96
262	EC929T	F	64	I	TRS	*TP53* mutation	2	Positive	High	Dead	1.54
263	EC927T	M	60	IIB	TRS	*TP53* mutation	1	Negative	Low	Dead	1.99
264	EC1003T	M	64	III	TRS	*TP53* wild-type	0	Positive	High	Dead	1.78
265	EC038T	M	68	IIA	WES	*TP53* mutation	1	Negative	Low	Dead	1.47
266	EC930T	F	58	IIA	TRS	*TP53* mutation	1	Positive	High	Alive	4.91
267	EC931T	F	57	IIA	TRS	*TP53* wild-type	0	Negative	Low	Alive	4.63
268	EC1000T	M	67	IIA	TRS	*TP53* mutation	2	Positive	Low	Alive	4.64
269	EC999T	F	75	IIB	TRS	*TP53* mutation	1	Positive	Low	Alive	4.64
270	EC1001T	F	68	III	TRS	*TP53* mutation	2	Positive	High	Dead	1.92
271	G017_T	F	67	III	WES	*TP53* mutation	1	Negative	Low	Alive	4.70
272	EC894T	M	66	IIA	TRS	*TP53* mutation	1	Positive	High	Alive	4.68
273	EC887T	M	77	IIA	TRS	*TP53* wild-type	0	Negative	Low	Alive	4.68
274	EC895T	M	73	III	TRS	*TP53* mutation	1	Positive	High	Dead	2.11
275	EC890T	F	64	IIA	TRS	*TP53* mutation	1	Negative	Low	Alive	4.69
276	EC036T	M	49	I	WES	*TP53* wild-type	0	Positive	High	Alive	7.88
277	EC027T	M	47	IIA	WES	*TP53* mutation	1	Positive	Low	Alive	11.89
278	TW258BF	M	40	IIA	WES	*TP53* mutation	1	Not detected	Not detected	Dead	1.39
279	EC114T	M	64	III	WES	*TP53* mutation	1	Not detected	Not detected	Alive	10.78
280	EC099T	M	58	IIA	WES	*TP53* mutation	1	Not detected	Not detected	Dead	1.76
281	TW1375BF	M	55	I	WES	*TP53* mutation	1	Not detected	Not detected	Alive	9.72
282	EC067T	M	50	IVB	WES	*TP53* wild*-*type	0	Not detected	Not detected	Dead	1.98
283	EC107T	M	56	III	WES	*TP53* mutation	1	Not detected	Not detected	Dead	3.06
284	MC23T	F	59	III	WES	*TP53* wild-type	0	Not detected	Not detected	Dead	0.06
285	EC188T	M	64	IIA	WES	*TP53* mutation	1	Not detected	Not detected	Dead	1.93
286	MC27T	M	56	IIB	WES	*TP53* wild-type	0	Not detected	Not detected	Dead	1.99
287	EC101T	M	73	IIA	WES	*TP53* wild-type	0	Not detected	Not detected	Dead	2.51
288	EC081T	F	68	IIB	WES	*TP53* mutation	1	Not detected	Not detected	Dead	2.62
289	MC30T	F	41	IIB	WES	*TP53* wild-type	0	Not detected	Not detected	Alive	8.52
290	EC145T	M	51	III	WES	*TP53* mutation	1	Not detected	Not detected	Alive	2.88
291	EC131T	M	49	IVA	WES	*TP53* mutation	2	Not detected	Not detected	Alive	12.35
292	TW470BF	F	49	III	WES	*TP53* mutation	1	Not detected	Not detected	Alive	15.36
293	EC086T	M	36	IIA	WES	*TP53* mutation	1	Not detected	Not detected	Alive	11.00
294	EC013T	F	69	IIA	WES	*TP53* wild-type	0	Not detected	Not detected	Dead	2.92
295	EC126T	F	63	III	WES	*TP53* mutation	1	Not detected	Not detected	Alive	11.37
296	EC040T	M	59	III	WES	*TP53* mutation	2	Not detected	Not detected	Alive	8.24
297	TW1371BF	M	44	IIA	WES	*TP53* mutation	1	Not detected	Not detected	Dead	2.22
298	TW1371AF	M	43	IIB	WES	*TP53* mutation	1	Not detected	Not detected	Dead	0.92
299	MC34T	M	51	IIA	WES	*TP53* mutation	1	Not detected	Not detected	Dead	7.54
300	EC925T	M	59	IIB	TRS	*TP53* wild-type	0	Not detected	Not detected	Dead	1.77
301	CE2T	M	54	III	WES	*TP53* mutation	1	Not detected	Not detected	Alive	13.37
302	MC29T	M	60	IIB	WES	*TP53* mutation	1	Not detected	Not detected	Dead	4.26
303	EC034T	M	62	IIB	WES	*TP53* mutation	1	Not detected	Not detected	Dead	6.17
304	MC4T	M	69	IIA	WES	*TP53* wild-type	0	Not detected	Not detected	Dead	2.81
305	TW2309BF	F	45	IIA	WES	*TP53* mutation	1	Not detected	Not detected	Alive	11.74
306	EC128T	F	51	III	WES	*TP53* mutation	1	Not detected	Not detected	Dead	4.00
307	EC192T	M	57	III	WES	*TP53* mutation	1	Not detected	Not detected	Dead	2.78
308	EC061T	M	76	IIA	WES	*TP53* wild-type	0	Not detected	Not detected	Dead	5.83
309	TW002BF	M	59	I	WES	*TP53* wild-type	0	Not detected	Not detected	Alive	10.24
310	MC26T	M	44	III	WES	*TP53* mutation	1	Not detected	Not detected	Dead	1.66
311	EC060T	M	50	IIA	WES	*TP53* mutation	1	Not detected	Not detected	Alive	6.87
312	TW829AF	F	60	IIA	WES	*TP53* mutation	1	Not detected	Not detected	Alive	14.09
313	TW1099AF	M	48	IIA	WES	*TP53* mutation	2	Not detected	Not detected	Alive	11.02
314	EC094T	F	58	IIA	WES	*TP53* mutation	2	Not detected	Not detected	Dead	5.70
315	EC165T	M	55	III	WES	*TP53* wild-type	0	Not detected	Not detected	Dead	9.95
316	EC012T	F	51	IIA	WES	*TP53* mutation	1	Not detected	Not detected	Dead	1.36

**Table 4:** Descriptive features and functional effects of *TP53* mutated in ESCC patients.

No	Patient ID	Number of mutations	Mutations	Mutation type	exon	Mutation annotation (cDNA)	TP53 mutation (protein)	TA class	Align GVGD	p53 IHC (positive/negative)	p53 IHC (low/high)	Survival status	Survival time (years)
2	CE18T	1	7577509C > A	Nonsense	exon7	c.G772T	p.E258X	—	—	Negative	Low	Dead	4.73
4	CE4T	1	7578212G > A	Nonsense	exon6	c.C637T	p.R213X	—	—	Negative	Low	Dead	3.95
5	CE7T	1	7579528C > T	Nonsense	exon4	c.G159A	p.W53X	—	—	Positive	Low	Dead	5.00
6	CE15T	1	7578461C > A	Missense	exon5	c.G469T	p.V157F	Not-functional	C0	Positive	Low	Dead	5.81
8	CE20T	1	7578413C > T	Missense	exon5	c.G517A	p.V173M	Not-functional	C15	Positive	Low	Dead	4.07
9	CE26T	2	7577556C > A	Missense	exon7	c.G725T	p.C242F	Not-functional	C65	Positive	High	Dead	6.16
			7578406C > T	Missense	exon5	c.G524A	p.R175H	Not-functional	C25				
10	CE5T	1	7577105G > A	Missense	exon8	c.C833T	p.P278L	Not-functional	C65	Positive	Low	Dead	5.11
12	CE24T	1	7577539G > A	Missense	exon7	c.C742T	p.R248W	Not-functional	C65	Positive	High	Alive	11.04
13	CE21T	2	7577603G > C	Silent	exon7	c.C678G	p.G226G	—	—	Negative	Low	Dead	6.50
			7577610T > A	Splice_site	exon8	c.673-2A > T	—	—	—				
14	CE27T	1	7578406C > T	Missense	exon5	c.G524A	p.R175H	Not-functional	C25	Positive	High	Dead	2.41
16	EC889T	1	7577156C > A	Splice_site	exon9	c.783-1G > T	—	—	—	Positive	High	Dead	0.79
18	EC892T	1	7579529C > T	Nonsense	exon4	c.G158A	p.W53X	—	—	Positive	High	Dead	3.15
19	EC893T	1	7578406C > T	Missense	exon5	c.G524A	p.R175H	Not-functional	C25	Positive	High	Alive	4.71
20	EC1038T	1	7579414C > T	Nonsense	exon4	c.G273A	p.W91X	—	—	Positive	Low	Dead	1.49
22	EC1032T	1	7578403C > A	Missense	exon5	c.G527T	p.C176F	Partially functional	C65	Positive	High	Dead	2.27
23	EC632T	1	7577085C > T	Missense	exon8	c.G853A	p.E285K	Not-functional	C55	Positive	High	Alive	4.78
24	EC636T	1	7579691TGTCCTTACCA > T	Frame_Shift_Del	exon3	c.95_96del	p.L32fs	—	—	Negative	Low	Alive	4.77
25	EC090T	1	7578268A > C	Missense	exon6	c.T581G	p.L194R	Not-functional	C65	Positive	High	Alive	1.41
26	EC633T	1	7577022G > A	Nonsense	exon8	c.C916T	p.R306X	—	—	Positive	Low	Alive	4.80
27	EC640T	1	7579539T > TG	Frame_Shift_Ins	exon4	c.147_148insC	p.I50fs	—	—	Negative	Low	Alive	4.81
28	EC643T	1	7578235T > C	Missense	exon6	c.A614G	p.Y205C	Not-functional	C65	Positive	Low	Dead	2.54
29	EC644T	1	7579310A > G	Splice_site	exon5	c.375+2T > *C*	—	—	—	Negative	Low	Dead	2.33
30	EC641T	1	7578188C > A	Nonsense	exon6	c.G661T	p.E221X	—	—	Negative	Low	Alive	4.84
31	EC967T	2	7576897G > A	Nonsense	exon9	c.C949T	p.Q317X	—	—	Negative	Low	Alive	4.84
			7579494T > A	Nonsense	exon4	c.A193T	p.R65X	—	—				
32	EC968T	1	7577511A > C	Missense	exon7	c.T770G	p.L257R	Not-functional	C65	Positive	Low	Alive	4.85
33	EC966T	2	7574029C > T	Missense	exon10	c.G998A	p.R333H	Functional	C0	Positive	High	Dead	2.37
			7577580T > C	Missense	exon7	c.A701G	p.Y234C	Not-functional	C45				
34	EC965T	1	7578478G > C	Missense	exon5	c.C452G	p.P151R	Not-functional	C65	Positive	High	Dead	0.95
35	EC1024T	1	7578395G > A	Missense	exon5	c.C535T	p.H179Y	Partially functional	C65	Positive	Low	Dead	4.03
36	EC190T	2	7578520A > T	Missense	exon5	c.T410A	p.L137Q	Functional	C65	Positive	Low	Alive	10.80
			7579313G > A	Missense	exon4	c.C374T	p.T125M	Not-functional	C65				
37	EC969T	1	7577539G > A	Missense	exon7	c.C742T	p.R248W	Not-functional	C65	Positive	High	Alive	4.88
39	EC971T	1	7579311C > T	Splice_Site	exon5	c.375+1G > *A*	—	—	—	Negative	Low	Alive	5.21
40	EC973T	1	7579415C > T	Nonsense	exon4	c.G272A	p.W91X	—	—	Negative	Low	Dead	0.88
41	EC975T	1	7577058C > A	Nonsense	exon8	c.G880T	p.E294X	—	—	Positive	Low	Alive	4.88
42	EC976T	1	7577575A > G	Missense	exon7	c.T706C	p.Y236H	Not-functional	C35	Positive	Low	Dead	1.89
43	EC978T	1	7579722C > T	Splice_Site	exon4	c.75-1G > *A*	—	—	—	Negative	Low	Alive	5.26
44	EC981T	1	7578370C > T	Splice_Site	exon6	c.559+1G > *A*	—	—	—	Negative	Low	Alive	4.90
45	EC982T	1	7577105G > C	Missense	exon8	c.C833G	p.P278R	Not-functional	C65	Positive	Low	Alive	4.90
46	EC983T	1	7574003G > A	Nonsense	exon10	c.C1024T	p.R342X	—	—	Negative	Low	Alive	3.95
47	EC191T	1	7578412A > G	Missense	exon5	c.T518C	p.V173A	Partially functional	C55	Positive	Low	Dead	4.18
48	EC984T	1	7578466G > T	Missense	exon5	c.C464A	p.T155N	Not-functional	C0	Positive	Low	Dead	1.36
49	EC985T	1	7577511A > G	Missense	exon7	c.T770C	p.L257P	Not-functional	C65	Negative	Low	Alive	5.30
50	EC986T	1	7578275G > A	Nonsense	exon6	c.C574T	p.Q192X	—	—	Negative	Low	Dead	3.24
51	EC987T	1	7577018C > T	Splice_Site	exon9	c.919+1G > *A*	—	—	—	Positive	Low	Alive	4.94
52	EC990T	1	7577539G > A	Missense	exon7	c.C742T	p.R248W	Not-functional	C65	Negative	Low	Dead	1.64
53	EC991T	1	7577503AGTCTTCCAGT > A	Frame_Shift_Del	exon7	c.768_777del	p.T256fs	—	—	Positive	Low	Dead	2.42
55	EC992T	1	7577094G > A	Missense	exon8	c.C844T	p.R282W	Not-functional	C65	Positive	Low	Alive	4.96
56	EC994T	1	7578394T > C	Missense	exon5	c.A536G	p.H179R	Not-functional	C25	Positive	Low	Alive	2.81
58	EC088T	1	7578469CCGGG > C	Frame_Shift_Del	exon5	c.457_460del	p.P153fs	—	—	Negative	Low	Dead	6.07
59	EC996T	1	7578403C > A	Missense	exon5	c.G527T	p.C176F	Partially functional	C65	Positive	High	Alive	4.98
60	EC630T	1	7577153C > A	Missense	exon8	c.G785T	p.G262V	Not-functional	C65	Positive	High	Dead	2.79
61	EC997T	1	7578406C > T	Missense	exon5	c.G524A	p.R175H	Not-functional	C25	Positive	Low	Alive	4.98
62	EC629T	1	7577156C > T	Splice_Site	exon9	c.783-1G > *A*	—	—	—	Positive	Low	Dead	2.43
63	EC1006T	2	7577106G > T	Missense	exon8	c.C832A	p.P278T	Not-functional	C35	Positive	High	Alive	5.04
			7578475G > A	Missense	exon5	c.C455T	p.P152L	Not-functional	C65				
64	EC885T	2	7577548C > T	Missense	exon7	c.G733A	p.G245S	Not-functional	C55	Positive	Low	Dead	4.01
			7576888T > A	Nonsense	exon9	c.A958T	p.K320X	—	—				
65	EC1005T	1	7578536T > C	Missense	exon5	c.A394G	p.K132E	Not-functional	C55	Positive	Low	Dead	2.92
67	G008_T	1	7578406C > T	Missense	exon5	c.G524A	p.R175H	Not-functional	C25	Positive	High	Dead	2.53
68	NR131225_2T	1	7578290C > T	Splice_Site	exon7	c.560-1G > *A*	—	—	—	Negative	Low	Alive	5.29
69	EC080T	1	7577143CAGT > C	non_Frame_Del	exon8	c.792_794del	p.264_265del	—	—	Positive	Low	Dead	2.70
70	NR131225_3T	1	7577082C > T	Missense	exon8	c.G856A	p.E286K	Not-functional	C55	Positive	High	Alive	5.32
71	NR131225_5T	1	7576870C > A	Nonsense	exon9	c.G976T	p.E326X	—	—	Negative	Low	Dead	0.93
72	NR131225_9T	1	7578239C > A	Nonsense	exon6	c.G610T	p.E204X	—	—	Negative	Low	Alive	5.67
73	NR131225_4T	1	7577120C > T	Missense	exon8	c.G818A	p.R273H	Not-functional	C25	Positive	Low	Dead	3.61
75	NR131225_1T	1	7578403C > A	Missense	exon5	c.G527T	p.C176F	Partially functional	C65	Positive	High	Dead	3.70
77	NR131225_6T	1	7577120C > A	Missense	exon8	c.G818T	p.R273L	Not-functional	C65	Positive	High	Dead	2.83
78	NR131121_10T	1	7577565T > C	Missense	exon7	c.A716G	p.N239S	Not-functional	C45	Positive	High	Dead	0.66
79	NR131121_9T	1	7577538C > T	Missense	exon7	c.G743A	p.R248Q	Not-functional	C35	Positive	High	Dead	1.00
81	NR131121_8T	1	7577120C > T	Missense	exon8	c.G818A	p.R273H	Not-functional	C25	Positive	Low	Dead	4.00
82	NR131121_7T	1	7579355A > T	Missense	exon4	c.T332A	p.L111Q	Not-functional	C65	Positive	Low	Dead	5.27
83	NR131121_6T	1	7577142C > G	Missense	exon8	c.G796C	p.G266R	Not-functional	C65	Positive	High	Dead	1.01
84	NR131121_5T	1	7577575A > C	Missense	exon7	c.T706G	p.Y236D	Not-functional	C55	Positive	Low	Alive	4.80
85	NR131121_4T	1	7577117A > C	Missense	exon8	c.T821G	p.V274G	Not-functional	C65	Positive	High	Dead	0.58
86	NR131121_3T	1	7577046C > A	Nonsense	exon8	c.G892T	p.E298X	—	—	Positive	Low	Alive	5.57
87	NR131121_2T	2	7578446T > A	Missense	exon5	c.A484T	p.I162F	Not-functional	C0	Positive	High	Dead	4.49
			7578484G > T	Missense	exon5	c.C446A	p.S149Y	Functional	C0				
88	NR131121_1T	1	7577586A > T	Missense	exon7	c.T695A	p.I232N	Not-functional	C35	Positive	High	Dead	2.03
89	TW2352AF	1	7578208T > C	Missense	exon6	c.A641G	p.H214R	Not-functional	C0	Positive	Low	Alive	7.87
90	TW2351BF	1	7577085C > T	Missense	exon8	c.G853A	p.E285K	Not-functional	C55	Positive	High	Alive	8.28
91	EC062T	1	7578392C > A	Nonsense	exon5	c.G538T	p.E180X	—	—	Positive	Low	Dead	6.49
92	EC129T	1	7578212G > A	Nonsense	exon6	c.C637T	p.R213X	—	—	Negative	Low	Alive	10.30
93	EC127T	1	7578239C > A	Nonsense	exon6	c.G610T	p.E204X	—	—	Negative	Low	Dead	8.45
94	MC16T	1	7577609C > T	Splice_Site	exon8	c.673-1G > *A*	—	—	—	Negative	Low	Dead	7.80
95	MC17T	2	7578469C > A	Missense	exon5	c.G461T	p.G154V	Not-functional	C65	Positive	Low	Dead	6.87
			7579312C > T	Silent	exon4	c.G375A	p.T125T	—	—				
96	MC13T	1	7577090C > G	Missense	exon8	c.G848C	p.R283P	Not-functional	C35	Positive	High	Dead	4.25
97	MC7T	2	7577106G > A	Missense	exon8	c.C832T	p.P278S	Not-functional	C65	Positive	High	Dead	0.75
			7577538C > G	Missense	exon7	c.G743C	p.R248P	Not-functional	C65				
98	TW258AF	1	7578406C > T	Missense	exon5	c.G524A	p.R175H	Not-functional	C25	Positive	High	Dead	0.60
99	FE0008T	1	7577094G > A	Missense	exon8	c.C844T	p.R282W	Not-functional	C65	Positive	High	Dead	0.83
100	EC123T	1	7573991C > A	Nonsense	exon10	c.G1036T	p.E346X	—	—	Positive	Low	Dead	4.06
101	G556T	1	7579419AG > A	Frame_Shift_Del	exon4	c.267delC	p.P89fs	—	—	Positive	Low	Alive	2.65
102	G406T	1	7577118C > A	Missense	exon8	c.G820T	p.V274F	Not-functional	C45	Positive	High	Dead	0.46
103	FG0067T	2	7578449C > T	Missense	exon5	c.G481A	p.A161T	Partially functional	C55	Positive	High	Alive	1.10
			7579362A > C	Missense	exon4	c.T325G	p.F109V	Not-functional	C0				
105	G414T	1	7578524G > A	Nonsense	exon5	c.C406T	p.Q136X	—	—	Negative	Low	Alive	3.11
106	EC727T	1	7577121G > A	Missense	exon8	c.C817T	p.R273C	Not-functional	C65	Positive	High	Alive	3.14
107	EC720T	1	7578275G > A	Nonsense	exon6	c.C574T	p.Q192X	—	—	Negative	Low	Dead	1.16
109	EC721T	1	7578203C > T	Missense	exon6	c.G646A	p.V216M	Not-functional	C15	Positive	High	Dead	0.48
110	EC943T	1	7578206T > C	Missense	exon6	c.A643G	p.S215G	Not-functional	C0	Positive	High	Alive	3.79
111	EC121T	1	7578271T > G	Missense	exon6	c.A578C	p.H193P	Not-functional	C65	Positive	High	Dead	1.00
112	EC854T	2	7577124C > A	Missense	exon8	c.G814T	p.V272L	Not-functional	C25	Positive	High	Alive	3.44
			7578290C > G	Splice_Site	exon7	c.560-1G > *C*	—	—	—				
113	EC868T	1	7577120C > T	Missense	exon8	c.G818A	p.R273H	Not-functional	C25	Positive	Low	Alive	3.44
114	EC686T	2	7577124C > A	Missense	exon8	c.G814T	p.V272L	Not-functional	C25	Positive	Low	Alive	3.43
			7578275G > A	Nonsense	exon6	c.C574T	p.Q192X	—	—				
118	EC883T	2	7577539G > A	Missense	exon7	c.C742T	p.R248W	Not-functional	C65	Positive	High	Alive	3.49
			7578192G > GGGCACCACCACACTATGTC	Frame_Shift_Ins	exon6	c.656_657insGACATAGTGTGGTGGTGCC	p.P219_Y220delinsPTX	—	—				
119	EC881T	1	7577153C > A	Missense	exon8	c.G785T	p.G262V	Not-functional	C65	Positive	High	Dead	1.24
120	EC877T	1	7578413C > A	Missense	exon5	c.G517T	p.V173L	Not-functional	C25	Positive	High	Alive	3.51
121	EC880T	1	7578443A > G	Missense	exon5	c.T487C	p.Y163H	Not-functional	C65	Positive	High	Alive	3.47
122	EC122T	1	7579485C > A	Nonsense	exon4	c.G202T	p.E68X	—	—	Negative	Low	Alive	11.47
124	EC871T	1	7577094G > A	Missense	exon8	c.C844T	p.R282W	Not-functional	C65	Positive	High	Alive	3.51
125	EC874T	1	7578211CGA > C	Frame_Shift_Del	exon6	c.636_637del	p.F212fs	—	—	Negative	Low	Alive	3.53
128	EC872T	1	7577094G > A	Missense	exon8	c.C844T	p.R282W	Not-functional	C65	Positive	High	Dead	0.89
129	EC875T	1	7574003G > A	Nonsense	exon10	c.C1024T	p.R342X	—	—	Positive	Low	Dead	1.99
130	EC869T	1	7577106G > A	Missense	exon8	c.C832T	p.P278S	Not-functional	C65	Positive	High	Dead	1.53
132	G479T	1	7578263G > A	Nonsense	exon6	c.C586T	p.R196X	—	—	Positive	Low	Dead	0.57
133	EC124T	1	7577569A > T	Missense	exon7	c.T712A	p.C238S	Not-functional	C65	Positive	High	Dead	2.07
134	EC814T	1	7579591C > A	Splice_Site	exon5	c.97-1G > *T*	—	—	—	Negative	Low	Alive	3.58
135	EC813T	1	7578212G > A	Nonsense	exon6	c.C637T	p.R213X	—	—	Positive	High	Alive	3.58
138	EC822T	1	7578190T > C	Missense	exon6	c.A659G	p.Y220C	Not-functional	C65	Positive	Low	Alive	3.95
139	EC818T	2	7577094G > A	Missense	exon8	c.C844T	p.R282W	Not-functional	C65	Positive	Low	Alive	3.58
			7578246CAA > C	Frame_Shift_Del	exon6	c.601_602del	p.L201fs	—	—				
140	EC821T	1	7577538C > T	Missense	exon7	c.G743A	p.R248Q	Not-functional	C35	Positive	High	Alive	3.59
142	EC832T	1	7577610T > C	Splice_Site	exon8	c.673-2A > *G*	—	—	—	Negative	Low	Dead	0.25
143	EC851T	1	7578212G > A	Nonsense	exon6	c.C637T	p.R213X	—	—	Negative	Low	Alive	3.64
144	EC120T	1	7577120C > T	Missense	exon8	c.G818A	p.R273H	Not-functional	C25	Positive	High	Dead	3.49
145	EC835T	1	7578221TTC > T	Frame_Shift_Del	exon6	c.626_627del	p.R209fs	—	—	Negative	Low	Dead	1.26
146	EC834T	1	7579360G > GA	Frame_Shift_Ins	exon4	c.326dupT	p.F109fs	—	—	Negative	Low	Alive	3.66
147	EC859T	1	7577058C > A	Nonsense	exon8	c.G880T	p.E294X	—	—	Negative	Low	Dead	1.02
149	EC838T	1	7577570C > T	Missense	exon7	c.G711A	p.M237I	Not-functional	C0	Positive	High	Alive	2.67
150	EC849T	1	7578394T > C	Missense	exon5	c.A536G	p.H179R	Not-functional	C25	Positive	High	Dead	1.26
151	EC848T	1	7578266TAAGATGCTG > T	non_Frame_Del	exon6	c.574_582del	p.192_194del	—	—	Positive	High	Alive	3.73
152	EC847T	1	7578263G > A	Nonsense	exon6	c.C586T	p.R196X	—	—	Negative	Low	Alive	3.72
153	EC845T	1	7578371C > T	Missense	exon5	c.G559A	p.G187S	Functional	C0	Negative	Low	Alive	3.75
154	EC932T	1	7579400G > GAA	Frame_Shift_Ins	exon4	c.286_287insTT	p.S96fs	—	—	Negative	Low	Dead	2.10
155	EC116T	1	7577094G > A	Missense	exon8	c.C844T	p.R282W	Not-functional	C65	Positive	High	Alive	11.53
158	EC933T	1	7578440T > A	Nonsense	exon5	c.A490T	p.K164X	—	—	Negative	Low	Dead	3.20
159	EC941T	1	7577609C > G	Splice_Site	exon8	c.673-1G > *C*	—	—	—	Negative	Low	Dead	2.57
160	EC942T	1	7577149AT > A	Frame_Shift_Del	exon8	c.788delA	p.N263fs	—	—	Negative	Low	Alive	3.78
161	EC945T	1	7577082C > T	Missense	exon8	c.G856A	p.E286K	Not-functional	C55	Positive	High	Alive	3.80
162	EC944T	1	7577539G > A	Missense	exon7	c.C742T	p.R248W	Not-functional	C65	Positive	High	Dead	1.31
165	EC948T	1	7576927C > T	Splice_Site	exon10	c.920-1G > *A*	—	—	—	Negative	Low	Alive	3.81
166	EC117T	3	7577556C > A	Missense	exon7	c.G725T	p.C242F	Not-functional	C65	Positive	High	Dead	1.03
			7577558G > A	Silent	exon7	c.C723T	p.S241S	—	—				
			7578406C > T	Missense	exon5	c.G524A	p.R175H	Not-functional	C25				
167	EC146T	1	7578395G > A	Missense	exon5	c.C535T	p.H179Y	Partially functional	C65	Positive	Low	Dead	3.25
168	EC940T	1	7579591C > G	Splice_Site	exon5	c.97-1G > *C*	—	—	—	Negative	Low	Dead	3.09
171	EC951T	2	7577144A > C	Missense	exon8	c.T794G	p.L265R	Not-functional	C65	Positive	High	Alive	3.85
			7578446T > TGGC	non_Frame_Ins	exon5	c.483_484insGCC	p.I162delinsAI	—	—				
172	EC952T	1	7579699C > T	Splice_Site	exon4	c.96+1G > *A*	—	—	—	Negative	Low	Alive	3.85
173	EC963T	2	7576927C > T	Splice_Site	exon10	c.920-1G > *A*	—	—	—	Negative	Low	Alive	3.86
			7578212G > A	Nonsense	exon6	c.C637T	p.R213X	—	—				
174	EC958T	1	7579470C > CG	Frame_Shift_Ins	exon4	c.216dupC	p.V73fs	—	—	Negative	Low	Alive	3.86
176	EC961T	2	7577121G > A	Missense	exon8	c.C817T	p.R273C	Not-functional	C65	Positive	High	Alive	3.88
			7578263G > A	Nonsense	exon6	c.C586T	p.R196X	—	—				
177	EC962T	1	7574029CG > C	Frame_Shift_Del	exon10	c.997delC	p.R333fs	—	—	Positive	Low	Alive	4.24
178	EC118T	1	7578212G > A	Nonsense	exon6	c.C637T	p.R213X	—	—	Negative	Low	Dead	3.05
180	EC960T	2	7578242C > A	Missense	exon6	c.G607T	p.V203L	Partially functional	C0	Positive	High	Dead	1.98
			7576865A > C	Nonsense	exon9	c.T981G	p.Y327X	—	—				
182	EC955T	1	7578249AT > A	Frame_Shift_Del	exon6	c.599delA	p.N200fs	—	—	Negative	Low	Dead	1.20
184	EC802T	1	7578368CA > C	Splice_Site InDel	exon6	c.559+2T > -	—	—	—	Negative	Low	Dead	1.48
185	EC793T	1	7577610T > G	Splice_Site	exon8	c.673-2A > *C*	—	—	—	Negative	Low	Alive	3.95
186	EC789T	1	7576927C > A	Splice_Site	exon10	c.920-1G > *T*	—	—	—	Negative	Low	Alive	3.95
187	EC797T	1	7578176C > T	Splice_Site	exon7	c.672+1G > *A*	—	—	—	Negative	Low	Alive	4.33
188	EC798T	1	7576928T > C	Splice_Site	exon10	c.920-2A > *G*	—	—	—	Negative	Low	Alive	3.96
189	EC115T	2	7577105G > A	Missense	exon8	c.C833T	p.P278L	Not-functional	C65	Positive	High	Dead	1.48
			7578538T > A	Missense	exon5	c.A392T	p.N131I	Not-functional	C65				
191	EC792T	1	7574029CG > C	Frame_Shift_Del	exon10	c.997delC	p.R333fs	—	—	Positive	Low	Dead	3.21
192	EC791T	3	7578215A > C	Missense	exon6	c.T634G	p.F212V	Partially functional	C0	Positive	High	Dead	1.25
			7577022G > A	Nonsense	exon8	c.C916T	p.R306X	—	—				
			7579699C > A	Splice_Site	exon4	c.96+1G > *T*	—	—	—				
193	EC790T	1	7578419C > A	Nonsense	exon5	c.G511T	p.E171X	—	—	Negative	Low	Dead	1.78
194	EC794T	2	7578406C > T	Missense	exon5	c.G524A	p.R175H	Not-functional	C25	Positive	High	Dead	1.74
			7578370CCATCGCTATCTGAGCAG > C	Frame_Shift_Del	exon5	c.543_559del	p.R181fs	—	—				
195	EC783T	1	7577121G > C	Missense	exon8	c.C817G	p.R273G	Not-functional	C65	Positive	High	Alive	3.97
197	EC795T	1	7578388C > G	Missense	exon5	c.G542C	p.R181P	Not-functional	C65	Positive	High	Dead	1.51
198	EC787T	1	7577547C > T	Missense	exon7	c.G734A	p.G245D	Not-functional	C65	Positive	High	Dead	0.84
199	G521T	1	7579311C > A	Splice_Site	exon5	c.375+1G > *T*	—	—	—	Negative	Low	Alive	3.63
200	EC113T	2	7577518T > A	Missense	exon7	c.A763T	p.I255F	Not-functional	C15	Positive	Low	Alive	11.54
			7578212G > A	Nonsense	exon6	c.C637T	p.R213X	—	—				
201	EC780T	1	7577093CG > C	Frame_Shift_Del	exon8	c.844delC	p.R282fs	—	—	Negative	Low	Dead	1.96
202	EC786T	1	7578271T > C	Missense	exon6	c.A578G	p.H193R	Not-functional	C65	Positive	High	Alive	4.05
203	EC781T	1	7578524G > C	Missense	exon5	c.C406G	p.Q136E	Not-functional	C25	Positive	High	Dead	1.33
205	EC779T	1	7577120C > T	Missense	exon8	c.G818A	p.R273H	Not-functional	C25	Positive	High	Alive	4.42
206	EC766T	1	7577035TG > T	Frame_Shift_Del	exon8	c.902delC	p.P301fs	—	—	Positive	Low	Dead	1.61
207	EC769T	1	7577106G > T	Missense	exon8	c.C832A	p.P278T	Not-functional	C35	Positive	High	Alive	4.46
208	EC807T	1	7578454G > A	Missense	exon5	c.C476T	p.A159V	Not-functional	C0	Positive	High	Alive	4.11
209	EC900T	1	7579414C > T	Nonsense	exon4	c.G273A	p.W91X	—	—	Negative	Low	Alive	4.11
210	EC898T	1	7578212G > A	Nonsense	exon6	c.C637T	p.R213X	—	—	Negative	Low	Alive	4.13
212	EC904T	1	7577142C > T	Missense	exon8	c.G796A	p.G266R	Not-functional	C65	Positive	High	Alive	4.12
214	EC908T	1	7574018G > A	Missense	exon10	c.C1009T	p.R337C	Not-functional	C45	Positive	High	Dead	1.88
215	EC907T	1	7577124C > A	Missense	exon8	c.G814T	p.V272L	Not-functional	C25	Positive	High	Dead	15.80
217	EC902T	1	7577094G > C	Missense	exon8	c.C844G	p.R282G	Not-functional	C65	Positive	Low	Alive	4.15
218	EC906T	1	7578437G > A	Nonsense	exon5	c.C493T	p.Q165X	—	—	Not detected	Not detected	Alive	4.51
222	TW094BF	2	7576857A > C	Missense	exon9	c.T989G	p.L330R	Not-functional	C65	Positive	Low	Dead	2.74
			7578176C > A	Splice_Site	exon7	c.672 + 1G > *T*	—	—	—				
223	G042_T	1	7578427T > C	Missense	exon5	c.A503G	p.H168R	Not-functional	C25	Negative	Low	Alive	4.16
226	EC909T	1	7578177C > T	Silent	exon6	c.G672A	p.E224E	—	—	Negative	Low	Alive	3.12
227	EC916T	1	7578395G > A	Missense	exon5	c.C535T	p.H179Y	Partially functional	C65	Positive	Low	Alive	4.18
229	EC913T	1	7578249AT > A	Frame_Shift_Del	exon6	c.599delA	p.N200fs	—	—	Negative	Low	Dead	3.05
231	EC920T	1	7578442T > C	Missense	exon5	c.A488G	p.Y163C	Not-functional	C65	Positive	High	Dead	2.15
232	EC918T	1	7578406C > T	Missense	exon5	c.G524A	p.R175H	Not-functional	C25	Positive	High	Dead	2.21
233	TW890BF	1	7577121G > A	Missense	exon8	c.C817T	p.R273C	Not-functional	C65	Positive	Low	Dead	2.35
236	EC921T	1	7579592T > A	Splice_Site	exon5	c.97-2A > *T*	—	—	—	Negative	Low	Dead	2.00
238	EC924T	1	7577121G > A	Missense	exon8	c.C817T	p.R273C	Not-functional	C65	Positive	High	Dead	3.30
240	EC684T	1	7578223CT > C	Frame_Shift_Del	exon6	c.625delA	p.R209fs	—	—	Negative	Low	Dead	3.84
241	EC682T	1	7577152AC > A	Frame_Shift_Del	exon8	c.785delG	p.G262fs	—	—	Negative	Low	Alive	4.42
243	EC680T	1	7579419AG > A	Frame_Shift_Del	exon4	c.267delC	p.P89fs	—	—	Negative	Low	Alive	4.42
244	EC055T	1	7577124C > T	Missense	exon8	c.G814A	p.V272M	Not-functional	C15	Positive	High	Dead	0.88
245	EC681T	1	7577610T > A	Splice_Site	exon8	c.673-2A > *T*	—	—	—	Negative	Low	Alive	4.44
247	EC676T	1	7579311C > T	Splice_Site	exon5	c.375+1G > *A*	—	—	—	Negative	Low	Dead	0.98
248	EC677T	1	7576851A > G	Splice_Site	exon10	c.993+2T > *C*	—	—	—	Negative	Low	Alive	4.44
249	EC672T	1	7576851A > G	Splice_Site	exon10	c.993+2T > *C*	—	—	—	Negative	Low	Dead	1.83
250	EC666T	1	7578212G > A	Nonsense	exon6	c.C637T	p.R213X	—	—	Negative	Low	Dead	1.54
251	EC660T	1	7579358C > A	Missense	exon4	c.G329T	p.R110L	Not-functional	C25	Positive	High	Alive	4.50
253	EC659T	1	7578479G > A	Missense	exon5	c.C451T	p.P151S	Not-functional	C65	Positive	High	Alive	4.87
254	EC1002T	1	7577094G > A	Missense	exon8	c.C844T	p.R282W	Not-functional	C65	Positive	High	Alive	4.65
258	EC651T	1	7578413C > A	Missense	exon5	c.G517T	p.V173L	Not-functional	C25	Positive	High	Alive	4.53
259	EC645T	1	7579528C > T	Nonsense	exon4	c.G159A	p.W53X	—	—	Negative	Low	Alive	4.55
260	EC928T	2	7577141C > T	Missense	exon8	c.G797A	p.G266E	Not-functional	C65	Positive	Low	Alive	4.58
			7578263G > A	Nonsense	exon6	c.C586T	p.R196X	—	—				
261	EC926T	1	7578265A > AT	Frame_Shift_Ins	exon6	c.583dupA	p.I195fs	—	—	Negative	Low	Alive	4.96
262	EC929T	2	7579355A > G	Missense	exon4	c.T332C	p.L111P	Not-functional	C65	Positive	High	Dead	1.54
			7579354C > A	Silent	exon4	c.G333T	p.L111L	—	—				
263	EC927T	1	7578175A > G	Splice_Site	exon7	c.672+2T > *C*	—	—	—	Negative	Low	Dead	1.99
265	EC038T	1	7579335TC > T	Frame_Shift_Del	exon4	c.351delG	p.G117fs	—	—	Negative	Low	Dead	1.47
266	EC930T	1	7578265A > T	Missense	exon6	c.T584A	p.I195N	Not-functional	C65	Positive	High	Alive	4.91
268	EC1000T	2	7577105G > C	Missense	exon8	c.C833G	p.P278R	Not-functional	C65	Positive	Low	Alive	4.64
			7578406C > T	Missense	exon5	c.G524A	p.R175H	Not-functional	C25				
269	EC999T	1	7578469C > A	Missense	exon5	c.G461T	p.G154V	Not-functional	C65	Positive	Low	Alive	4.64
270	EC1001T	2	7578413C > T	Missense	exon5	c.G517A	p.V173M	Not-functional	C15	Positive	High	Dead	1.92
			7578263G > A	Nonsense	exon6	c.C586T	p.R196X	—	—				
271	G017_T	1	7578212G > A	Nonsense	exon6	c.C637T	p.R213X	—	—	Negative	Low	Alive	4.70
272	EC894T	1	7577094G > A	Missense	exon8	c.C844T	p.R282W	Not-functional	C65	Positive	High	Alive	4.68
274	EC895T	1	7578265A > G	Missense	exon6	c.T584C	p.I195T	Not-functional	C55	Positive	High	Dead	2.11
275	EC890T	1	7578245G > GC	Frame_Shift_Ins	exon6	c.603dupG	p.R202fs	—	—	Negative	Low	Alive	4.69
277	EC027T	1	7578526C > A	Missense	exon5	c.G404T	p.C135F	Not-functional	C65	Positive	Low	Alive	11.89
278	TW258BF	1	7577609C > T	Splice_Site	exon8	c.673-1G > *A*	—	—	—	Not detected	Not detected	Dead	1.39
279	EC114T	1	7577144A > C	Missense	exon8	c.T794G	p.L265R	Not-functional	C65	Not detected	Not detected	Alive	10.78
280	EC099T	1	7578384G > T	Nonsense	exon5	c.C546A	p.C182X	—	—	Not detected	Not detected	Dead	1.76
281	TW1375BF	1	7578263G > A	Nonsense	exon6	c.C586T	p.R196X	—	—	Not detected	Not detected	Alive	9.72
283	EC107T	1	7579388T > TG	Frame_Shift_Ins	exon4	c.298dupC	p.Q100fs	—	—	Not detected	Not detected	Dead	3.06
285	EC188T	1	7579315G > GC	Frame_Shift_Ins	exon4	c.371dupG	p.C124fs	—	—	Not detected	Not detected	Dead	1.93
288	EC081T	1	7578265A > G	Missense	exon6	c.T584C	p.I195T	Not-functional	C55	Not detected	Not detected	Dead	2.62
290	EC145T	1	7578413C > T	Missense	exon5	c.G517A	p.V173M	Not-functional	C15	Not detected	Not detected	Alive	2.88
291	EC131T	2	7578413C > A	Missense	exon5	c.G517T	p.V173L	Not-functional	C25	Not detected	Not detected	Alive	12.35
			7574000C > A	Nonsense	exon10	c.G1027T	p.E343X	—	—				
292	TW470BF	1	7577544A > T	Missense	exon7	c.T737A	p.M246K	Not-functional	C65	Not detected	Not detected	Alive	15.36
293	EC086T	1	7579372GC > G	Frame_Shift_Del	exon4	c.314delG	p.G105fs	—	—	Not detected	Not detected	Alive	11.00
295	EC126T	1	7579470CG > C	Frame_Shift_Del	exon4	c.216delC	p.P72fs	—	—	Not detected	Not detected	Alive	11.37
296	EC040T	2	7577568C > G	Missense	exon7	c.G713C	p.C238S	Not-functional	C65	Not detected	Not detected	Alive	8.24
			7577022G > A	Nonsense	exon8	c.C916T	p.R306X	—	—				
297	TW1371BF	1	7578483GGAAT > G	Frame_Shift_Del	exon5	c.443_446del	p.D148fs	—	—	Not detected	Not detected	Dead	2.22
298	TW1371AF	1	7578271T > A	Missense	exon6	c.A578T	p.H193L	Not-functional	C65	Not detected	Not detected	Dead	0.92
299	MC34T	1	7577538C > A	Missense	exon7	c.G743T	p.R248L	Not-functional	C65	Not detected	Not detected	Dead	7.54
301	CE2T	1	7578205CTA > C	Frame_Shift_Del	exon6	c.642_643del	p.H214fs	—	—	Not detected	Not detected	Alive	13.37
302	MC29T	1	7579345CA > C	Frame_Shift_Del	exon4	c.341delT	p.L114fs	—	—	Not detected	Not detected	Dead	4.26
303	EC034T	1	7577539G > A	Missense	exon7	c.C742T	p.R248W	Not-functional	C65	Not detected	Not detected	Dead	6.17
305	TW2309BF	1	7577022G > A	Nonsense	exon8	c.C916T	p.R306X	—	—	Not detected	Not detected	Alive	11.74
306	EC128T	1	7578406C > T	Missense	exon5	c.G524A	p.R175H	Not-functional	C25	Not detected	Not detected	Dead	4.00
307	EC192T	1	7578395G > A	Missense	exon5	c.C535T	p.H179Y	Partially functional	C65	Not detected	Not detected	Dead	2.78
310	MC26T	1	7578265A > G	Missense	exon6	c.T584C	p.I195T	Not-functional	C55	Not detected	Not detected	Dead	1.66
311	EC060T	1	7578179C > A	Nonsense	exon6	c.G670T	p.E224X	—	—	Not detected	Not detected	Alive	6.87
312	TW829AF	1	7579315G > GC	Frame_Shift_Ins	exon4	c.371dupG	p.C124fs	—	—	Not detected	Not detected	Alive	14.09
313	TW1099AF	2	7578208T > C	Missense	exon6	c.A641G	p.H214R	Not-functional	C0	Not detected	Not detected	Alive	11.02
			7579485C > A	Nonsense	exon4	c.G202T	p.E68X	—	—				
314	EC094T	2	7577539G > A	Missense	exon7	c.C742T	p.R248W	Not-functional	C65	Not detected	Not detected	Dead	5.70
			7576928T > A	Splice_Site	exon10	c.920-2A > *T*	—	—	—				
316	EC012T	1	7579315G > GC	Frame_Shift_Ins	exon4	c.371dupG	p.C124fs	—	—	Not detected	Not detected	Dead	1.36

**Table 5:** Association between *TP53* mutation and p53 protein expression in 276 ESCC patients.

p53 protein expression	*TP53* Mutation				*TP53*wild-type, *n* = 64
	Missense, *n* = 121	Nonsense, *n* = 36	Others, *n* = 55	Total	
p53 high/low expression					
p53 high expression	81	2	2	85	23
p53 low expression	40	34	53	127	41
p53 positive/negative expression					
p53 expression (+)	117	11	10	138	38
p53 expression (−)	4	25	45	74	26

Note. When a missense mutation occurs, patients with multiple mutations are preferentially included in the missense mutation group. When a nonsense mutation occurs, the remaining patients are preferentially included in the nonsense mutation group.

**Table 6:** Association between p53 protein expression and clinicopathologic features in patients with ESCC in tissue microarray.

Characteristics	All patients,	p53 protein expression				*p* value
		Low expression,		High expression,		
	*n* = 6028	*n* = 4209	(%)	*n* = 1819	(%)	
Sex						0.525
Female	2107	1482	70.3	625	29.7	
Male	3921	2727	69.5	1194	30.5	
Age at diagnosis						0.492
≤60	2917	2049	70.2	868	29.8	
>60	3111	2160	69.4	951	30.6	
High/low incidence area						0.003
Low	2119	1531	72.3	588	27.7	
High	3909	2678	68.5	1231	31.5	
Cigarette smoking						0.393
Negative	3160	2217	70.2	943	29.8	
Positive	2731	1888	69.1	843	30.9	
Alcohol consumption						0.901
Negative	4103	2857	69.6	1246	30.4	
Positive	1791	1250	69.8	541	30.2	
Family history						0.615
Negative	3875	2703	69.8	1172	30.2	
Positive	2037	1408	69.1	629	30.9	
Location						0.667
Cervical + upper	954	678	71.1	276	28.9	
Middle	4092	2842	69.5	1250	30.5	
Lower	879	613	69.7	266	30.3	
Mix	51	33	64.7	18	35.3	
Differentiation						<0.001
Well differentiated	501	385	76.8	116	23.2	
Moderate differentiated	3303	2350	71.1	953	28.9	
Poor differentiated	1935	1280	66.1	655	33.9	
Pathological T stage						0.335
Tis + T1	205	134	65.4	71	34.6	
T2	1466	1022	69.7	444	30.3	
T3	4242	2978	70.2	1264	29.8	
T4	115	75	65.2	40	34.8	
Pathological N stage						0.494
N0	3354	2354	70.2	1000	29.8	
N1	2674	1855	69.4	819	30.6	
Pathological M stage						0.930
M0	5814	4059	69.8	1755	30.2	
M1	214	150	70.1	64	29.9	
UICC stage (6th)						0.144
0 + I	144	89	61.8	55	38.2	
II	3691	2600	70.4	1091	29.6	
III	1979	1370	69.2	609	30.8	
IV	214	150	70.1	64	29.9	
Cancer embolus						0.028
Negative	5772	4046	70.1	1726	29.9	
Positive	256	163	63.7	93	36.3	
Type of treatment						0.596
Surgical	5006	3499	69.9	1507	30.1	
Surgical + chemo	493	352	71.4	141	28.6	
Surgical + radio	395	269	68.1	126	31.9	
Surgical + chemo + radio	134	89	66.4	45	33.6	

## Data Availability

The data that support the findings of this study are available from the corresponding author upon reasonable request.
